# Sequence based residue depth prediction using evolutionary information and predicted secondary structure

**DOI:** 10.1186/1471-2105-9-388

**Published:** 2008-09-20

**Authors:** Hua Zhang, Tuo Zhang, Ke Chen, Shiyi Shen, Jishou Ruan, Lukasz Kurgan

**Affiliations:** 1 College of Mathematical Science and LPMC, Nankai University, Tianjin, PR China; 2 Department of Electrical and Computer Engineering, University of Alberta, Edmonton, AB, Canada; 3 Chern Institute of Mathematics, Tianjin, PR China

## Abstract

**Background:**

Residue depth allows determining how deeply a given residue is buried, in contrast to the solvent accessibility that differentiates between buried and solvent-exposed residues. When compared with the solvent accessibility, the depth allows studying deep-level structures and functional sites, and formation of the protein folding nucleus. Accurate prediction of residue depth would provide valuable information for fold recognition, prediction of functional sites, and protein design.

**Results:**

A new method, RDPred, for the real-value depth prediction from protein sequence is proposed. RDPred combines information extracted from the sequence, PSI-BLAST scoring matrices, and secondary structure predicted with PSIPRED. Three-fold/ten-fold cross validation based tests performed on three independent, low-identity datasets show that the distance based depth (computed using MSMS) predicted by RDPred is characterized by 0.67/0.67, 0.66/0.67, and 0.64/0.65 correlation with the actual depth, by the mean absolute errors equal 0.56/0.56, 0.61/0.60, and 0.58/0.57, and by the mean relative errors equal 17.0%/16.9%, 18.2%/18.1%, and 17.7%/17.6%, respectively. The mean absolute and the mean relative errors are shown to be statistically significantly better when compared with a method recently proposed by Yuan and Wang [Proteins 2008; 70:509–516]. The results show that three-fold cross validation underestimates the variability of the prediction quality when compared with the results based on the ten-fold cross validation. We also show that the hydrophilic and flexible residues are predicted more accurately than hydrophobic and rigid residues. Similarly, the charged residues that include Lys, Glu, Asp, and Arg are the most accurately predicted. Our analysis reveals that evolutionary information encoded using PSSM is characterized by stronger correlation with the depth for hydrophilic amino acids (AAs) and aliphatic AAs when compared with hydrophobic AAs and aromatic AAs. Finally, we show that the secondary structure of coils and strands is useful in depth prediction, in contrast to helices that have relatively uniform distribution over the protein depth. Application of the predicted residue depth to prediction of buried/exposed residues shows consistent improvements in detection rates of both buried and exposed residues when compared with the competing method. Finally, we contrasted the prediction performance among distance based (MSMS and DPX) and volume based (SADIC) depth definitions. We found that the distance based indices are harder to predict due to the more complex nature of the corresponding depth profiles.

**Conclusion:**

The proposed method, RDPred, provides statistically significantly better predictions of residue depth when compared with the competing method. The predicted depth can be used to provide improved prediction of both buried and exposed residues. The prediction of exposed residues has implications in characterization/prediction of interactions with ligands and other proteins, while the prediction of buried residues could be used in the context of folding predictions and simulations.

## Background

Knowledge of the tertiary (3D) protein structure is vital when addressing the problems in protein folding and function. The commonly accepted hypothesis that protein sequence uniquely determines protein structure [1] enables development of methods for prediction of 3D structure from sequence. Such methods are of substantial value due to the large and exponentially growing sequence-structure gap. Currently, the sequence based 3D structure prediction is still a challenging task [2,3]. Therefore, a set of intermediate, more tractable predictions that target various structural aspects, such as solvent-accessible surface area (ASA), secondary structure (SS), contact number or order, etc., were researched and applied to predict protein structure and function.

The residues that constitute a protein could be divided into surface residues and the remaining residues that are buried in the protein's interior. Since surface residues are directly involved in the interaction with other biological molecules, they have been widely studied [4,5] and used for identifying protein function and stability [6,7] and to aid fold recognition [8,9]. The prediction of the relative solvent accessibility (RSA), which is defined as the ASA of each residue in the protein divided by that observed in an extended (Gly-X-Gly or Ala-X-Ala) conformation and which can be used to identify surface residues, was addressed by a number of methods [10-17]. At the same time, the buried residues, which were shown to have similar local packing arrangements irrespective of protein size [18] also play important roles including formation of a hydrophobic core that helps maintaining protein folding conformation [19] and maintaining of the structural integrity of the protein due to their high degree of conservation that is also shown to have impact on formation of enzyme active sites [20], among others. However, ASA values that can be accurately predicted from protein sequence, e.g. Wang and colleagues reported 0.66 correlation between the predicted and the actual RSA values [17], cannot provide sufficient information to characterize buried residues, i.e. the ASA values of the buried residues are zero or near zero.

As an alternative, the depth of an atom or residue in the protein has been proposed to characterize spatial arrangement of protein structures [21-23]. Several definitions of atom depth have been proposed, including those distance (the minimum distance between an atom and a dot of solvent accessible surface [22] or its closest solvent accessible neighbor [23,24]) or volume dependant [25]. The residue depth (RD) is the average atom depth of all atoms composing this residue. This descriptor has been shown to have higher correlation with residue conservation when compared with ASA. It was also found to be useful for analysis of amide hydrogen/deuterium exchange rates in nuclear magnetic resonance (NMR) experiments [21], for analysis of local packing arrangements in the protein core [18], for analysis and prediction of function sites, such as catalytic sites of enzyme [26] and phosphorylation sites [23,27], and for protein fold recognition [9,28]. Recently, it has been suggested that the most deeply buried residues in the native protein fold might be the first to fold [29], which signifies their importance with respect to folding predictions and simulations. Based on the above, an accurate methodology to predict residue depth from the sequence would provide a useful input for analysis and designing methods for the protein folding.

To date, only one method for prediction of the residue depth based on the protein sequence was developed [30]. This method, which utilizes information encoded in the PSI-BLAST scoring matrix and a support vector regression (SVR) predictor [31], was designed and tested using a large dataset of 923 chains (YW923). This method is characterized by the correlation coefficient between the predicted and the actual depth and the mean absolute error of the depth prediction equal 0.65 and 0.60, respectively. The authors considered the position-specific scoring matrix generated by PSI-BLAST and protein size as the only inputs, although other information could be used to further improve the predictions. In our work, we focus on adding additional inputs (features) that are based on the predicted secondary structure, position in sequence and information per position from PSI-BLAST to provide higher quality residue depth predictions. Our careful design of the proposed method, which is based on performing feature selection and parameterization of the SVR predictor, is also shown to contribute to the improvements. We show that the proposed method provides statistically significantly better predictions when compared with the competing method by Yuan and Wang [30]. We also analyze the selected features to discuss factors related to the residue depth, and we show that the application of the proposed method to prediction of surface/buried residues also shows improvements when compared with the competing method.

## Methods

**Figure 1:**
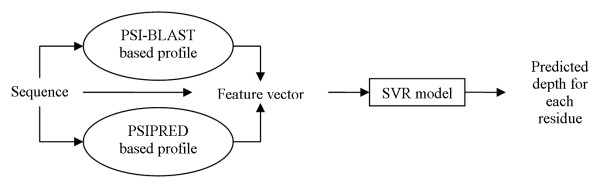
Proposed prediction system.

The sequence based prediction methods work in two steps: (1) a fixed-length feature vector is computed based on the sequence and sequence-derived information; (2) the vector is inputted into the prediction model to generate the outcome. The design of the prediction method usually requires a training dataset that is used to construct the model and parameterize the algorithm used to generate the model. In our case we choose SVR to construct the model due to its extensive prior use in bioinformatics application that perform real-valued prediction [12,32-35], including the existing sequence based depth prediction [30]. More specifically, given a sequence, the features were extracted from the sequence itself, and from the sequence-derived PSI-BLAST scoring matrix [36] and secondary structure predicted with PSIPRED [37,38], see Figure 1.

### Datasets

We prepared four datasets to test and design the proposed method. The first is the original dataset, YW923, used to design and test method proposed by Yuan and Wang [30]. This dataset is composed of 923 protein chains including X-ray and NMR structures culled using PDB-PRERDB [39] with 25% sequence identity threshold. The included structures were solved by X-ray crystallography with resolution ≤ 2.0 Å and R-factor ≤ 0.2. All NMR structures for those datasets are selected from the first model in PDB files.

Following the test procedure performed in [30], we adopted three-fold cross-validation in our design and tests with YW923 dataset. Due to the large size of the dataset, which imposes time consuming parameterization of SVR, we created a subset of the original dataset that includes randomly chosen 50 sequences from each fold created from the YW923. This dataset, which is composed of 150 chains, is referred to as YW150. The YW150 is used to parameterize SVR and to perform feature selection (see "Prediction Method" Section).

We also prepared two new datasets from the sequences that are deposited in the Protein Data Bank (PDB) [40] between Jan 2007 and Aug 2007, which were filtered to have low identity with the sequences deposited before 2007. More specifically, the sequences deposited before 2007 were filtered using cd-hit program [41] with 90% identity threshold (this set is referred to as 90PDB). The sequences deposited between Jan 2007 and Aug 2007 were filtered with cd-hit with global 40% identity threshold (this set is referred to as 40PDB). Although cd-hit is a computationally efficient application, it is limited to the minimal threshold of 40%, and thus we used Needleman-Wunsch alignment algorithm [42] to filter out sequences deposited in 2007 that share above 25% identity to sequences from 90PDB. As a result, the first new dataset, called PDB491, includes 491 sequences that share at most 40% identity with each other, and at most 25% identity with sequences published in PDB before 2007. To reduce local sequence similarity that could be included when global alignment is used (i.e., when using CD-hit and Needleman-Wunsch algorithms), NCBI's BLASTCLUST program [34] was applied to the union set of 90PDB, YW923 and 40PDB, with the local identity threshold of 25% (-S 25) and default minimal length coverage of 90% (-L 0.9). The second new dataset was constructed by selecting one chain from each of the clusters that contained no sequences from 90PDB and YW923 datasets. This set, called PDB366, includes 366 sequences that, as a result, have local 25% identity with each other and also with both 90PDB and YW923. We note that both PDB491 and PDB366 share low identity (especially PDB366 dataset which includes sequences with low local similarity) with the YW923 dataset that was created before 2007, and also with the training set used by PSIPRED version 2.5 (which was updated in Apr 2006) that we applied to compute the predicted secondary structure. This allows us to perform tests on a dataset that is independent of the sequences used to design this method and other methods (such as PSIPRED) that were used to provide our inputs. The PDB ids of the sequences from both new datasets (PDB366 and PDB491) are included in the Additional file 1.

### Calculation of residue depth

We computed the residue depths for all above datasets following the procedure that had been used in [30]. First, MSMS program [43] was executed with a probe radius of 1.4 Å to obtain a list of vertices that represent protein surface. The atom depth of an atom, which is defined as the distance between the atom and the nearest vertex [44], was calculated. Finally, the average depth of all atoms except the hydrogen atoms in an amino acid defines the residue depth. All residue depth values in abovementioned datasets were normalized using the same mean depth ($\bar{X}$) and its standard deviation (*σ*) as follows

$$X_{norm}=\frac{X-\bar{X}}{\sigma }$$

where $\bar{X}$ = 2.64 Å and *σ *= 1.41 Å were derived from YW923 dataset as shown in [30]. For the new datasets, the corresponding mean depths and standard deviations are $\bar{X}$ = 2.73 Å and *σ *= 1.42 Å for PDB491 and $\bar{X}$ = 2.65 Å and *σ *= 1.32 Å for PDB366, respectively, which are similar to those values of YW923 dataset. Only the former values (i.e. mean depth and standard deviation from YW923 dataset) were used to normalize the actual depths of all four datasets in order to assure consistency when performing independent (blind) tests on PDB491 and PDB366 datasets.

In [30], only the abovementioned MSMS-based depth index has been considered. In order to analyze differences in prediction performance between distance and volume dependant depth indices, we also computed two other depth indices, DPX [23,24] and SADIC [25], for the YW923 dataset. To calculate DPX values, first, NACCESS program [45] was applied to obtain the solvent-accessible surface areas (ASA) for all atoms. Then the atom depth was calculated as the distance between a given atom and the nearest solvent accessible atom that had positive ASA. Similarly to MSMS-based depth calculation, the DPX value of a residue is the average atom depth of all its atoms except the hydrogen atoms. To compute SADIC-based index, given an atom *i *of a molecule and a sampling radius *r*, a depth index *D*_*i*, *r *_is defined as *D*_*i*, *r *_= 2*V*_*i*, *r*_*/V*_0, *r*_, where *V*_*i*, *r *_is the exposed volume of a sphere of radius *r *centered on atom *i *and *V*_0, *r *_is the exposed volume of the same sphere when centered on an isolated atom. Following [25], we computed this index values for *Cα *atoms as the residue depths with a sampling radius of 9 Å. Similarly as in the case of the MSMS-based depth, the residue depth values for two other indices in YW923 dataset were normalized according to their mean depths and standard deviations with $\bar{X}$ = 0.94 Å and *σ *= 1.05 Å for DPX and with $\bar{X}$ = 0.45 and *σ *= 0.33 for SADIC algorithm (the values are based on a ratio of two volumes and thus they have no unit) using the above formula.

### Feature Vector

The feature vector used by the proposed method was encoded based on global and local information that was obtained from three sources: sequence (sequence-based features), multiple alignment (PSI-BLAST-based features), and predicted secondary structure (PSIPRED-based features).

#### Sequence-based Features

Earlier contributions [22,23] have shown that protein size is correlated with both maximum and average residue depths, and this relationship could be represented by a linear or nearly linear function. Yuan and Wang also showed the beneficial effect of the protein size on the accuracy of depth prediction [30]. Hence, our method also includes this feature. We normalized this features by dividing the sequence length by 1000.

Additionally, we observe that the residues at the termini (for both C-terminus and N-terminus) are usually on the surface or close to the surface. We define the position of the *i*^th ^residue in the sequence as

$$position=\frac{\left|i-(L+1)/2\right|}{L/2}$$

where *L *is the sequence length. This value represents the sequence distance to the center of the chain. These two features (size and position) constitute our sequence-based features.

#### PSI-BLAST-based Features

Similarly as in PSIPRED [37], PSI-BLAST was used to perform multiple alignment of the input sequence with the E-value equal 10^-3 ^and three-iterations against the NCBI nonredundant protein sequence database (nr database); this database was filtered to remove low-complexity regions, transmembrane regions, and coiled-coil segments. PSI-BLAST's output includes position-specific scoring matrix (PSSM) and information per position (IPP). The PSSM is a *L *× 20 matrix, where 20 is the number of amino acid types. The score values are first normalized by using standard logistic function

$$\frac{1}{1+\exp(-x)}.$$

The information per position provides a quantitative measure of sequence conservation among the homologous sequences used to construct the PSSM for each position [46]. This value, which is stored in the second last column in PSI-BLAST profiles, is provided directly by PSI-BLAST.

Next, in our coding scheme, a sliding window of 15 neighboring residues was used to represent the evolutionary information of a sequence, where each position of the window had 21 possible values with 20 from PSSM and one from IPP. The selection of the window size is motivated by the size used in [30]. We denote the PSI-BLAST based features by PSSM_*α*_^*h *^and IPP^*h *^for each residue, where *α *represents a type of 20 amino acids and *h *= {-7, -6,.., +6, +7} represents a position in the sliding window (*h *= 0 corresponds to the central residue, *h *< 0 (> 0) corresponds to positions towards N-terminus (C-teminus) with distance of *h *from the central residue. As a result, there are 15 × 21 features generated from the PSI-BLAST's output.

#### PSIPRED-based Features

The secondary structure prediction was performed using PSIPRED due to the following: (1) PSIPRED was recently shown to provide superior accuracy when compared with other state-of-the-art secondary structure prediction methods[47]; (2) this method is frequently used to support a variety of other predictions tasks such as solvent accessibility [13], fold prediction [48], folding rate prediction [49], predictions of beta-turns [50], alpha-turns [51], contact order [34], to name just a few. The motivation to include secondary structure came from the differences in distributions of the residue depth values within to three secondary structures i.e. helix (H), strand (E), and coil (C) [30]. While Yuan and Wang considered the actual secondary structure to analyze the distributions [30], we apply the secondary structures predicted from sequences. We note that the PSIPRED predictions include the secondary structure state for each residue that is accompanied by the corresponding probability. Based on the insights provided in [52], we investigate whether these probabilities could be used to address residue depth prediction.

We designed several feature sets based on the outputs from the PSIPRED, which include local and global information. The local information is composed of 15 × 3 features that concern probabilities in a window of 15 neighboring residues, where the secondary structure of each residue is represented by a 3-dimensional probability vector, i.e., probability of coil, strand, and helix prediction. These secondary structure probability profiles (SSP) for each residue are denoted by SSP_*α*_^*h*^, h = {-7, -6,.., +6, +7} comprising 45 features. The global information is coded by the secondary structure content and the frequency of secondary structure segments as follows:

$$\begin{array}{cc} content_{\alpha }=\frac{\#\alpha }{\#H+\#E+\#C} & fseg_{\alpha }=\frac{\#seg_{\alpha }}{\#seg_{H}+\#seg_{E}+\#seg_{C}} \end{array}$$

where *α *= {H, E, C}, *#α *is the number of secondary structures of type *α *in the sequence, and *#seg*_*α *_is the number of segments that only contain one type of consecutive secondary structures *α*. We note that since one or two consecutive predicted helical residues could not form a helix segment, they are replaced by coils when computing the frequency of secondary structure segments. Hence, we obtain 15 × 3 + 3 + 3 = 51 PSIPRED-based features.

Overall, we produced 368 features that serve as the input vector for the proposed prediction model, see Table 1. In contrast, Yuan and Wang used 15 × 20 features based on the PSSM matrix and 1 feature based on the protein size (total of 301 features) [30]. For the sake of comparison with the results of Yuan and Wang's (YW) method [30], we combine the position and IPP features together (they correspond to information computed per position). This way, we define four sets of features that include PSSM (300 features computed from PSSM), SS (51 features that concern the probabilities of the secondary structure, the content, and the segment frequency), PS (protein size), and PI (position and information per position).

**Table 1:** Summary of the computed features.

Feature set type	Feature set name	# features
Sequence-based	Protein size	1
	Position	1
PSI-BLAST-based	PSSM_*α*_^*h*^	300
	IPP^*h*^	15
PSIPRED-based	SSP_*α*_^*h*^	45
	fseg_*α*_	3
	content_*α*_	3

### Prediction Method

#### Support Vector Regression

SVR is a regression model based on the support vector machine (SVM) [53]. The motivation behind the choice of SVR comes from wide-spread applications of SVRs in real-value predictions concerning various bioinformatics problems, such as prediction of accessible surface areas [12,15], contact numbers [32], protein B-factors [54], gene expression levels [33], contact orders [34], and finally the application in the existing method for residue depth prediction [30].

SVR predictor maps feature vectors into multi-dimensional space by using kernel function *K*, *ε*-insensitive loss function and regulatory parameter *C*. In our case, we use Guassian kernel function *K*(*x*_*i*_, *x*_*j*_) = exp (-*γ *||*x*_*i *_– *x*_*j*_||^2^). The choice of the kernel function is motivated by its competitive performance for solving nonlinear problems when compared with other kernel functions [54] and prior use for the residue depth prediction [30]. Similarly as in [30] the error tube parameter *ε *is set to equal 0.001, while we optimize the kernel parameter *γ *and regulatory parameter *C *through a grid search on YW150 dataset (see "Parameterization of SVR" section). A detailed description of SVR could be found in [53].

#### Evaluation of prediction performance

The performance of the proposed method was evaluated based on *n*-fold cross validation performed on YW923, PDB491, and PDB366 datasets. The protein chains were randomly divided into *n *subsets to create cross validation folds. We performed three-fold cross validation (3 CV) to maintain consistency with results reported in [30] and ten-fold cross validation (10 CV). Furthermore, we also performed blind tests by training the prediction model on the YW923 dataset and testing on two new datasets, PDB491 and PDB366.

**Figure 2:**
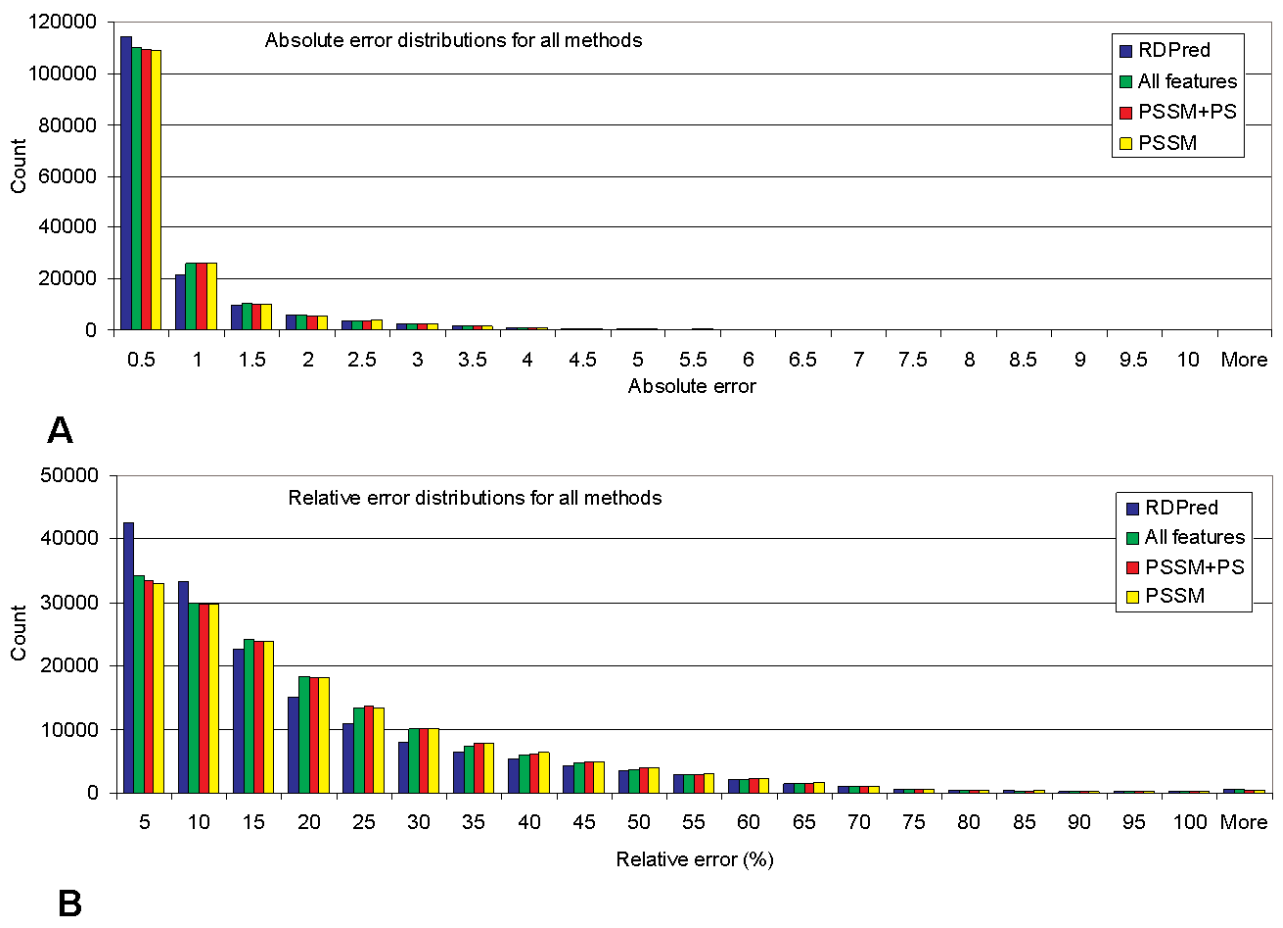
**The distributions of absolute errors and relative errors at the residue level for four prediction methods (using different feature sets) on YW923 dataset based on the three-fold cross validation.** In panel (A) the bars show the counts of residues with the absolute errors in a bin size of 0.5. In panel (B) the bars show the counts of residues with the relative errors in a bin size of 5%.

We adopted three indices to validate and compare quality of the proposed method: Pearson correlation coefficient (PCC), mean absolute error (MAE) and mean relative error (MRE) between predicted and observed depths, which are defined as

$$\begin{array}{ccc} PCC=\frac{\sum_{i=1}^{N}\left(x_{i}-\bar{x})(y_{i}-\bar{y}\right)}{\sqrt{\left[\sum_{i=1}^{N}{\left(x_{i}-\bar{x}\right)}^{2}][\sum_{i=1}^{N}{\left(y_{i}-\bar{y}\right)}^{2}\right]}}, & MAE=\frac{1}{N}\sum_{i=1}^{N}\left|x_{i}-y_{i}\right|, & MRE=\frac{1}{N}\sum_{i=1}^{N}\left|\frac{x_{i}-y_{i}}{x_{i}}\right|, \end{array}$$

where *x*_*i *_and *y*_*i *_are the observed and predicted depth values of the *i*^th ^residue in sequence, respectively, and $\bar{x}$ and $\bar{y}$ are the corresponding mean values, respectively. The PCC is also used to rank individual features with respect to their relation with the depth values. We emphasize that the prediction quality was reported at the residue level, i.e. all residues in a given dataset were collected together and one PCC value across all residues was computed, while the MAE and MRE values are computed as the average over all residues. Based on our observations, both absolute errors and relative errors between the actual and the predicted depth values have skewed distributions (see Figure 2). Therefore, as suggested in [55], we report two outer centiles, i.e., the 10^th ^and 90^th ^centiles, to strengthen the analysis of the prediction performance. They show differences between the actual and the predicted depth values for errors with low and high magnitudes (this applies to both absolute errors and relative errors for each residue). Similarly as in [13] we also estimate the quality of the prediction at the fold level. The PCC, MAE, and MRE are computed for each test fold and next they are averaged over all folds. In this case we also report standard deviations to estimate the spread of the prediction performance.

#### Parameterization of SVR

The parameterization of SVR was performed through a grid search over *γ *and *C *values based on three-fold cross-validation on YW150 dataset for the MSMS based depth. We considered *γ *and *C *values drawn from {0.01, 0.02, 0.03,..., 0.1} × {0.1, 0.3, 0.5,..., 1.9} grid. Two parameterizations were performed: (1) using the PSSM and PS features, which corresponds to the design of Yuan and Wang [30], we parameterized SVR used to perform feature selection (see "Feature Selection" section); and (2) using the features selected by the feature selection from the set of all 368 features considered in this study. The first parameterization resulted in *γ *= 0.02 and *C *= 1.5, while the second gave *γ *= 0.06 and *C *= 0.5. The latter set of parameters is applied to build the proposed prediction model.

#### Feature Selection

To address high dimensionality of the feature set, we processed it to remove irrelevant features. This may lead to more compact prediction model, ability to interpret the selected features, and potentially better prediction quality. We applied a correlation-based feature selection [56], which utilizes the Pearson correlation coefficient (PCC) values measured between each feature and the target values, to select best-performing subset of the original 368 features. In case when the correlation coefficient is close to zero, the corresponding feature is not correlated with the prediction outcome, and thus it could be considered irrelevant. More specifically, we computed the PCC between each feature and the target depth values in two steps. First, the PCC values were computed for each of the three folds in YW923 dataset. Second, the average PCCs over the three folds was calculated and the absolute values were used to rank the original set of 368 features. This cross-validation based selection allows for maintaining consistency with the experimental evaluation of the depth prediction quality. Next, we tested the quality of the depth prediction using SVR with *γ *= 0.02 and *C *= 1.5 based on three-fold cross-validation on YW150 dataset with top 50, 75, 100, 125,..., 368 features. The best results, i.e., PCC = 0.629, MAE = 0.610 Å, and MRE = 18.1%, were obtained with a set of 125 features, thus reducing the size of the original feature set by 66%.

## Results and Discussion

### Prediction performance for the MSMS based depth

The proposed prediction method was implemented using LIBSVM library [57]. The three-fold and ten-fold cross-validations were used to estimate the out-of-sample prediction quality on YW923, PDB491, and PDB366 datasets for MSMS based depth index (results for two other depth indices are shown in the next section). The proposed prediction method, referred to as *RDPred*, uses 125 features and Gaussian-kernel based SVR with *γ *= 0.06 and *C *= 0.5.

Table 2 shows the comparison of predictive performance measured at the residue level between RDPred and Yuan and Wang's (YW) method [30], as well as when different feature sets were used. In particular, we include tests when the input sequences are represented using only PSSM features, PSSM and PS features, all 368 features considered in this study. The first two cases allow for comparison with [30] where the same features were used, but parameterization of the SVR was different. The third case allows evaluating the value added of the performed feature selection and the second SVR parameterization round.

**Table 2:** Comparison of prediction performance at the residue level on four datasets (YW923, YW150, PDB491 and PDB366) between the proposed RDPred method, the YW method (Yuan and Wang, 2008), and when applying different feature sets and test types (best results are bolded for each dataset and each test type).

		YW923			YW150		
		
Test type	Method	PCC	MAE	MRE (%)	PCC	MAE	MRE (%)
3 CV	YW method	0.65	0.60	18.0	**--**	**--**	**--**
	PSSM^a^	0.64	0.60 (0.049,1.533)	18.9 (2.4,43.1)	0.58	0.66 (0.057,1.591)	20.6 (2.7,45.7)
	PSSM+PS^a^	0.66	0.59 (0.049,1.502)	18.7 (2.4,42.6)	0.61	0.65 (0.054,1.581)	20.4 (2.6,45.5)
	All features^a^	0.68	0.58 **(**0.048,1.461)	18.5 (2.3,42.1)	0.62	0.64 (0.056,1.555)	20.4 (2.7,45.4)
	**RDPred**^b^	0.67	**0.56 **(0.037,1.510)	**17.0 **(1.8,41.4)	0.63	**0.61 **(0.041,1.557)	**18.1 **(2.0,43.2)

10 CV^c^	PSSM+PS^a^	0.67	0.58 (0.048,0.496)	18.5 (2.3,42.3)	**--**	**--**	**--**
	**RDPred**^b^	0.67	**0.56 **(0.036,1.501)	**16.9 **(1.8,41.1)	**--**	**--**	**--**

		PDB491			PDB366		
		
Test type	Method	PCC	MAE	MRE (%)	PCC	MAE	MRE (%)

3 CV	YW method	**--**	**--**	**--**	**--**	**--**	**--**
	PSSM^a^	0.65	0.64 (0.054,1.644)	20.0 (2.6,44.8)	0.63	0.62 (0.053,1.560)	19.6 (2.6,44.0)
	PSSM+PS^a^	0.66	0.64 (0.054,1.625)	20.0 (2.6,44.6)	0.63	0.61 (0.053,1.539)	19.6 (2.6,43.8)
	All features^a^	0.68	0.62 (0.054,1.582)	19.7 (2.5,43.8)	0.65	0.60 (0.053,1.507)	19.4 (2.5,43.5)
	**RDPred**^b^	0.66	**0.61 **(0.041,1.630)	**18.2 **(2.0,43.3)	0.64	**0.58 **(0.040,1.556)	**17.7 **(2.0,42.3)

10 CV	PSSM+PS^a^	0.66	0.63 (0.053, 1.620)	19.7 (2.5,44.1)	0.64	0.61 (0.053,1.535)	19.4 (2.5,43.6)
	**RDPred**^b^	0.67	**0.60 **(0.040,1.631)	**18.1 **(2.0,43.1)	0.65	**0.57 **(0.039,1.546)	**17.6 **(1.9,42.2)

^a^The SVR parameters are *ε *= 0.001, *γ *= 0.02, and *C *= 1.5.

^b^The SVR parameters are *ε *= 0.001, *γ *= 0.06, and *C *= 0.5; 125 features were used.

^c^Due to extensive computational cost, in the case of the 10-fold cross validation only the results for PSSM+PS and RDPred methods were reported. The results on the YW150 dataset were not reported since this dataset was only used to perform parameterization on the proposed method.

The two values in the brackets are the 10^th ^centile and the 90^th ^centile of the absolute and relative errors.

For the three-fold cross validation the experiments show that RDPred consistently provides better predictions, i.e., it obtains the lowest MAE and MRE as well as the lowest 10^th ^centile values for the absolute error and the lowest 10^th ^and 90^th ^centiles values for the relative error. For the YW923 dataset, when compared to YW method, RDPred improved the MAE to 0.558 (from 0.6), the PCC to 0.67 (from 0.65), and MRE to 17% (from 18%). Although the PCC has decreased and the 90^th ^centile of the absolute error has increased after the feature selection for YW923 dataset (0.668 for RDPred vs. 0.681 when using all features), both MAE and MRE as well as the 10^th ^centile of the absolute error and the 10^th ^and 90^th ^centiles of the relative error have improved justifying the need for the feature selection.

Most importantly, the same differences are observed for the two new datasets, PDB491 and PDB366. Table 2 shows that RDPred is better than predictions based on PSSM and PS features (which correspond to the design of YW method), i.e., for PDB491 dataset, the PCC was improved from 0.659 to 0.664, MAE from 0.639 to 0.607, and MRE from 20% to 18.2%, and for PDB366 dataset, the PCC was improved from 0.634 to 0.639, MAE from 0.614 to 0.579, and MRE from 19.6% to 17.7%. This shows that the performed parameterization and feature selection did not overfit the YW923 dataset.

**Figure 3:**
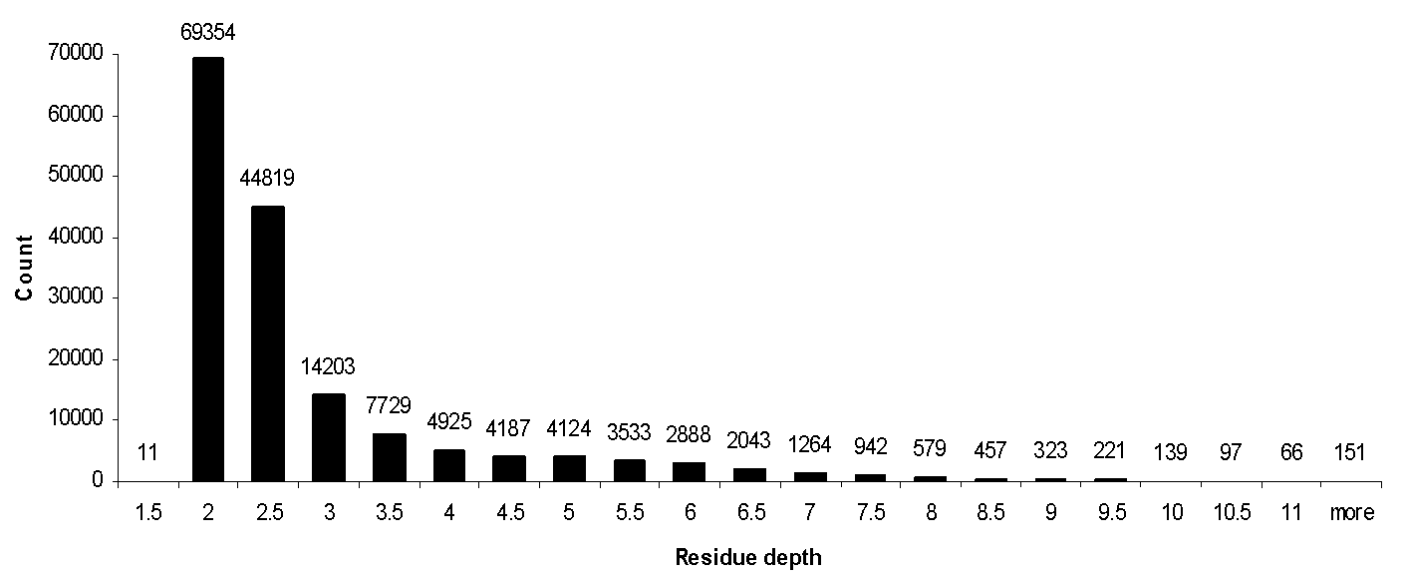
**The distribution of counts of residues at different depth level on YW923 dataset.** The depth values were binned to intervals of 0.5 size *(x*-axis), while the numbers above the bars shows the corresponding counts.

The (skewed) distributions of the absolute errors and the relative errors on YW923 dataset are shown in Figure 2. These distributions and the centile values from Table 2 show that RDPred generates more predictions with low error values and fewer predictions with high error values when compared with the other three methods. The skewness of errors is due to the skewed depth distribution, which is given in Figure 3.

**Figure 4:**
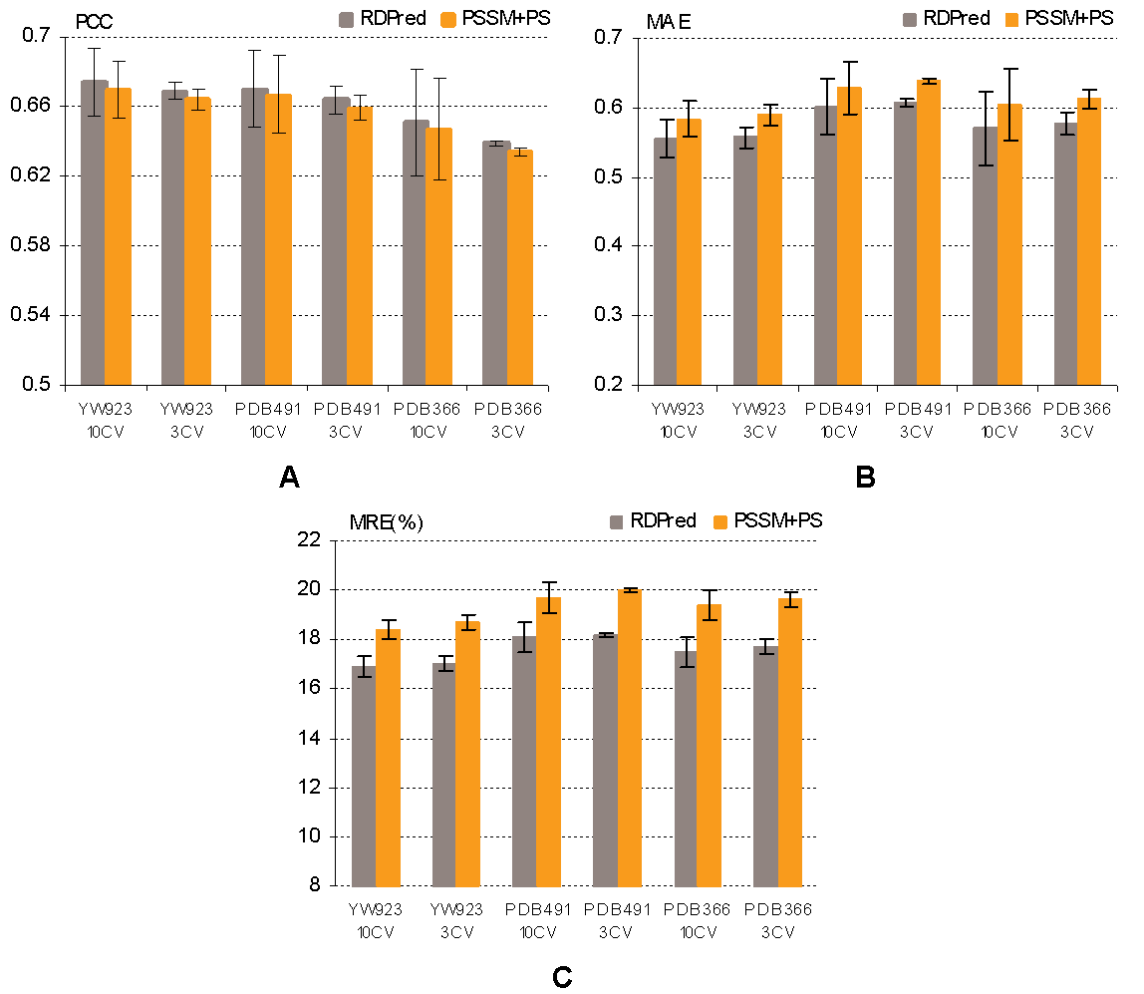
**The comparison of (A) PCC, (B) MAE and (C) MRE values at the fold level when using three-fold cross validation (3 CV) and ten-fold cross validation (10 CV) on the three datasets, i.e., YW923, PDB491 and PDB366.** The *x*-axis shows the corresponding datasets and test types, e.g., YW923 10 CV corresponds to the results on the YW923 dataset derived by ten-fold cross validation. The results are averaged over the folds and the corresponding standard deviations are shown using error bars. The scale of the *y*-axis, which shows the average quality index values, varies between the three panels.

Table 2 shows that the results based on the ten-fold cross validations for PSSM+PS and RDPred are consistent with corresponding results of the three-fold cross validation tests. Figure 4 compares the prediction performance at the fold level for RDPred and PSSM+PS using both three-fold and ten-fold cross validations for the three considered datasets. We observe that ten-fold cross validation results show larger error bars than the corresponding results obtained using three-fold cross-validation. This is due to a relatively large variability of the prediction quality for individual sequences. Overall, the Figure shows that although on average the PCC values are higher for the proposed RDPred method, the error bars show that the difference is not significant. On the other hand, the differences in the case of the MAE and MRE are more substantial. We performed the paired t-test at the 95% significance level for each type of the quality index, in which we compare the corresponding pairs of the ten-fold cross validation results on the three datasets. For datasets YW923, PDB491, and PDB366, the corresponding P-values for PCC equal 0.19, 0.22, and 0.21, respectively. The P-values for MAE and MRE were below 0.00000001 in the case of all three datasets. The t-test shows that the improvement with respect to PCC values is not significant; however, RDPred provides statistically significantly lower MAE and MRE values over all three datasets when compared with the predictions based on the PSSM+PS method.

**Figure 5:**
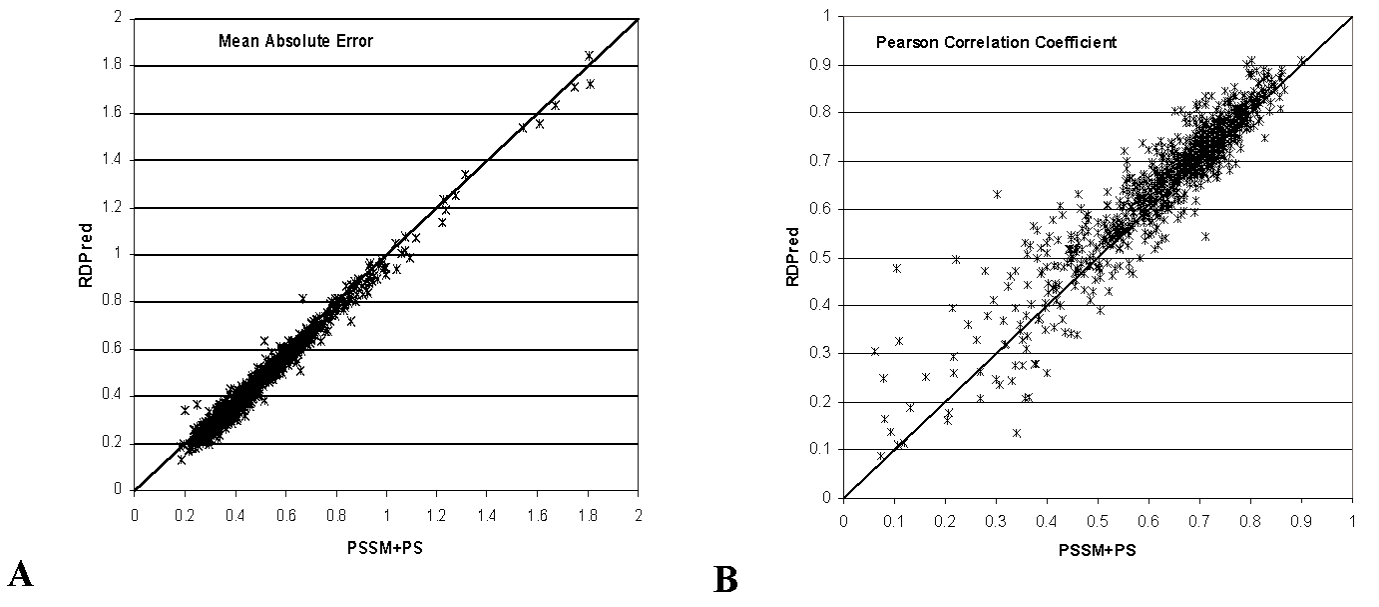
**The comparison between RDPred and the predictions based on PSSM and PS features (simulation of YW method).** Each point denotes prediction for one sequence in YW923 dataset. Panel (A) compares MAE values, while panel (B) compares PCC values; x-axis shows results for method based on based on PSSM and PS features; y-axis shows results for RDPred method.

We also performed a detailed, i.e., based on the predictions derived using three-fold cross validation for individual sequences in YW923 dataset, comparison between RDPred and YW method. Since the individual prediction were not available for YW method, we simulated their prediction by using PSSM and PS features with our SVR model (they used these exact features and SVR to perform predictions). These results allow evaluation of the value added of performing feature selection, adding SS (features based on predicted secondary stricture) and PI (position and information per position) features, and performing the SVR parameterization. Figure 5 shows the relations of MAE and PCC values between the RDPred and the simulation of YW method. In case of MAE, see Figure 5A, we observe that RDPred provides lower errors for majority of the predicted sequences, i.e., for 821 out of 923 proteins the RDPred predictions are below the diagonal which denotes points where both methods obtain equal errors. Similarly, for 646 out of 923 sequences, the RDPred gives higher PCC values; in this case the points are located above the diagonal, see Figure 5B. Furthermore, a paired t-test was applied to investigate statistical significance of these differences. The paired t-test performed at 95% significance level, which compared pairs of MAE values (and pairs of PCC values) for the same sequences predicted by RDPred and the simulation of the YW method, shows that in both cases, i.e., MAE and PCC, the RDPred provided statistically significantly better predictions. The corresponding P-values were smaller than 0.0001 for both PCC and MAE and t-values were equal 12.7 for PCC and 34.1 for MAE.

We also report and compare predictions of RDPred and the simulation of YW method that are binned with respect to the actual depth values, see Table 3. RDPred obtained better results for residue depth RD < 2.25 Å and RD ≥ 5.0 Å, similar results for 2.25 Å ≤ RD < 3.0 Å and 4.0 Å ≤ RD < 5.0 Å, and slightly worse predictions for 3.0 Å = RD < 4.0 Å when compared with the method based on PSSM+PS features. We observe relatively large improvement for shallow residues characterized by depth of below 2.25 Å, which constitute 61% of all residues. Similar degree of improvement is also observed for the deepest residue, i.e. those with depth above 5.0 Å. The 10^th ^centile values show that RDPred produces more predictions with smaller errors (the values are lower), while the 90^th ^centile shows that the proposed method obtains fewer larger errors (the values are also lower).

**Table 3:** Comparison of prediction errors for different depth bins for the RDPred and the simulation of YW method on YW923 dataset.

		PSSM+PS		RDPred	
		
Depth bin	# of residues	MAE	MRE (%)	MAE	MRE (%)
		0.25 (0.035,0.525)	14.8 (2.1,30.6)	0.19 (0.026,0.380)	11.0 (1.5,22.1)
1.75 ≤ RD < 2.0	56537	0.27 (0.034,0.604)	14.7 (1.8,32.6)	0.21 (0.021,0.474)	11.3 (1.2,25.5)
2.0 ≤ RD < 2.25	29172	0.33 (0.041,0.733)	15.6 (2.6,34.8)	0.29 (0.034,0.653)	13.7 (1.6,30.9)
2.25 ≤ RD < 2.5	15647	0.41 (0.061,0.845)	17.2 (2.6,35.7)	0.40 (0.062,0.794)	16.7 (2.6,33.4)
2.5 ≤ RD < 3.0	14203	0.51 (0.085,0.963)	18.6 (3.1,35.1)	0.53 (0.096,0.966)	19.6 (3.5,34.7)
3.0 ≤ RD < 4.0	12654	0.77 (0.153,1.490)	22.3 (4.5,40.2)	0.82 (0.170,1.495)	23.9 (5.0,41.8)
4.0 ≤ RD < 5.0	8311	1.32 (0.338,2.262)	29.1 (7.6,49.2)	1.33 (0.300,2.319)	29.4 (6.6,50.8)
5.0 ≤ RD < 6.0	6421	1.91 (0.643,3.041)	34.8 (11.8,55.3)	1.87 (0.497,3.127)	34.2 (9.0,57.1)
RD ≥ 6.0	6282	3.32 (1.419,5.35)	44.1 (21.0,64.7)	3.26 (1.158,5.386)	43.1 (17.1,65.8)

The values in the brackets correspond to the 10^th ^and 90^th ^centiles of the absolute and relative errors.

The improvements are attributed to the use of PSIPRED based features and the PI features (position and information per position). We note that YW923, PDB491, and PDB366 datasets are characterized by similar Q_3 _(3-state per-residue prediction accuracy) values for secondary structures predicted with PSIPRED (when compared with DSSP [58]), which equal 79.8%, 79.3% and 78.3% respectively. This shows that the improvements are due to the use of the proposed features rather than differences in the quality of input data (quality of predicted secondary structure).

To further strengthen the evaluation, we performed blind tests by training both PSSM+PS feature (simulation of YW method) and RDPred with YW923 dataset and testing with PDB491 and PDB366 datasets. The corresponding results are listed in Table 4. We observe an improvement due to the use of the proposed method, i.e., the MAE and MRE values together with the corresponding 10^th ^centile values are lower for both datasets in the case of RDPred, which is consistent with the results obtained based on the three fold cross validations shown in Table 2.

**Table 4:** Summary of the blind test results on PDB491 and PDB366 datasets

	PDB491			PDB366		
	
Method	PCC	MAE	MRE (%)	PCC	MAE	MRE (%)
PSSM+PS	0.663	0.638 (0.053,1.615)	20.4 (2.5,45.7)	0.650	0.601 (0.051,1.520)	19.8 (2.5,44.4)
RDPred	0.666	0.609 (0.041,1.635)	18.8 (2.0,44.4)	0.654	0.573 (0.038,1.534)	18.3 (1.9,43.2)

The prediction model was trained on YW923 dataset.

The values in the brackets correspond to the 10^th ^and 90^th ^centiles of the absolute and relative errors

The paired t-test performed at 95% significance level, which compares pairs of MAE values (and pairs of PCC values) for the same sequences predicted by RDPred and the simulation of the YW method, for the predictions from both blind tests shows that the differences are significant. More specifically, for the PDB366 dataset, P-values for both PCC and MAE were below 0.0001 while t-values were equal 7.5 and 18.4 for PCC and MAE, respectively. Similarly, for PDB491, the P-values were again below 0.0001 and t-values were equal 7.4 for PCC and 21.7 for MAE.

When comparing the results between the two datasets (YW923 and PDB491), higher MAE obtained for the PDB491 dataset shows that this datasets is more difficult (although consistent differences in quality between individual methods on each dataset are observed). One potential reason is that the PDB491 dataset is characterized by larger mean depth than that of YW923 (2.74 Å vs. 2.64 Å). This is also supported by smaller values of MAE for PDB366 dataset when compared with results on PDB491 dataset (see Tables 2 and 4) because of the lower mean depth of the former dataset (2.65 Å vs. 2.74 Å). As shown in (Yuan and Wang, 2008), buried residues are more difficult to predict than the exposed residues. Furthermore, PDB491 includes 277 multimer chains and 214 monomers while YW923 includes 250 multimer chains and 673 monomers. This shows that PDB491 has higher ratio of multimer chains, while these chains include larger number of buried residues. The poorer results on the YW150 dataset are due to the small size of this dataset.

**Figure 6:**
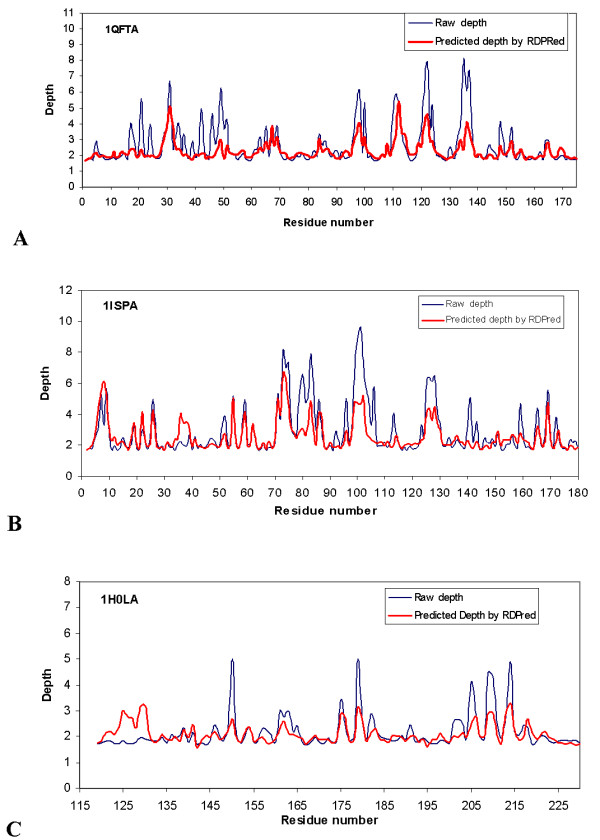
The observed and predicted depths by RDPred for three protein chains: Panel (A) 1QFTA; Panel (B) 1ISPA; and Panel (C) 1H0LA.

Figure 6 shows the observed depths and the depths predicted by RDPred for three representative protein chains, 1QFTA, 1ISPA and 1H0LA. The 1QFTA has MAE equal 0.555, which represents a prediction of average quality, see Figure 6A. The MAE of 1ISPA equals 0.637 (Figure 6B) and MAE of 1H0LA equals 0.317 (Figure 6C), which represents predictions with above average and below average quality, respectively. We observe that for all three cases, the depths of shallow (exposed) residues are predicted relatively well, i.e., their depths are neither under- or over-predicted. The main difference between the three typical prediction cases is the degree to which the deeply buried residues are predicted. In case of average or below average prediction, we observe that many of the buried residues are identified, but their depths are under-predicted. At the same time, Figure 6B that shows above average prediction indicates that depths of some of the buried residues are predicted accurately.

Table 5 lists the MAE for 20 amino acids (AAs), their mean depths and standard deviations of the depths in YW923 dataset, as well as several physicochemical properties including hydrophobic, charge, and flexibility indices that were obtained from the AA index database [59]. The rows are ordered in ascending order by the MAE values. We observe that predictions of RDPred result in smaller error for all amino acids types when compared with simulation of YW method (using PSSM and PS as the inputs); the only exception is Trp (W) in which case the same error value is obtained. This again shows consistency of the obtained improvement. The table also reveals that MAE obtained for each AA type is strongly correlated with its mean depth, standard deviation of the depth, hydrophobic scale values that quantify hydrophobic tendency of a given AA [60], and flexibility index [61]. The corresponding correlation coefficients for the RDPred's errors equal 0.98, 0.95, 0.89, and -0.90, respectively. We conclude the following: (a) AAs with smaller mean depths (and smaller standard deviations of the average depth) are easier (more accurate) to predict than those with the larger corresponding values, which implies that deeply buried residues are more difficult to correctly predict; (b) hydrophilic and flexible residues are more accurately predicted when compared with the hydrophobic and rigid residues; and (c) charged residues that include Lys (K), Glu (E), Asp (D), and Arg (R) are the easiest (the most accurate) to predict.

**Table 5:** The MAE, mean depth, standard deviation of the depth (stdev) in YW923 dataset for RDPred and simulation of YW method for the 20 amino acids (AAs).

	MAE					
					
AA	RDPred	PSSM+PS	Mean depth (± stdev)	Hydrophobicity	Charge	Flexibility
K	0.24	0.28				
E	0.28	0.33	2.05 (± 0.81)	-0.74	-1	1.094
D	0.33	0.37	2.08 (± 0.84)	-0.90	-1	1.068
R	0.34	0.37	2.24 (± 0.81)	-2.53	+1	1.008
Q	0.36	0.40	2.23 (± 0.97)	-0.85	0	1.037
N	0.40	0.43	2.26 (± 1.02)	-0.78	0	1.048
P	0.43	0.46	2.39 (± 1.08)	-0.12	0	1.049
S	0.47	0.51	2.38 (± 1.21)	-0.18	0	1.046
H	0.51	0.54	2.50 (± 1.19)	-0.4	+1	0.950
T	0.54	0.56	2.56 (± 1.32)	-0.05	0	0.997
G	0.58	0.62	2.43 (± 1.40)	0.48	0	1.031
A	0.64	0.68	2.77 (± 1.50)	0.62	0	0.984
Y	0.67	0.68	2.86 (± 1.30)	0.26	0	0.929
C	0.72	0.75	2.91 (± 1.39)	0.29	0	0.906
M	0.77	0.79	3.08 (± 1.58)	0.64	0	0.952
L	0.81	0.83	3.23 (± 1.53)	1.06	0	0.935
V	0.82	0.84	3.25 (± 1.59)	1.08	0	0.931
W	0.83	0.83	3.13 (± 1.43)	0.81	0	0.904
I	0.86	0.89	3.42 (± 1.64)	1.38	0	0.927
F	0.87	0.89	3.29 (± 1.58)	1.19	0	0.915

The table also includes hydrophobic index, charge, and flexibility index values for the 20 AAs.

### Predictions for different depth definitions

We computed the residue depths with DPX and SADIC algorithms on YW923 dataset and their correlations with each other and the MSMS based depth values are shown in Table 6. DPX is shown to be highly correlated with the MSMS-based values, while in the case of the SADIC algorithm, the correlation is lower but still relatively high.

**Table 6:** Correlation matrix for three depth definitions on dataset YW923.

	MSMS	DPX	SADIC
MSMS	1.0		
DPX	0.77	1.0	
SADIC	-0.63	-0.67	1.0

The matrix is symmetrical and thus the values in the upper triangle were left empty.

**Figure 7:**
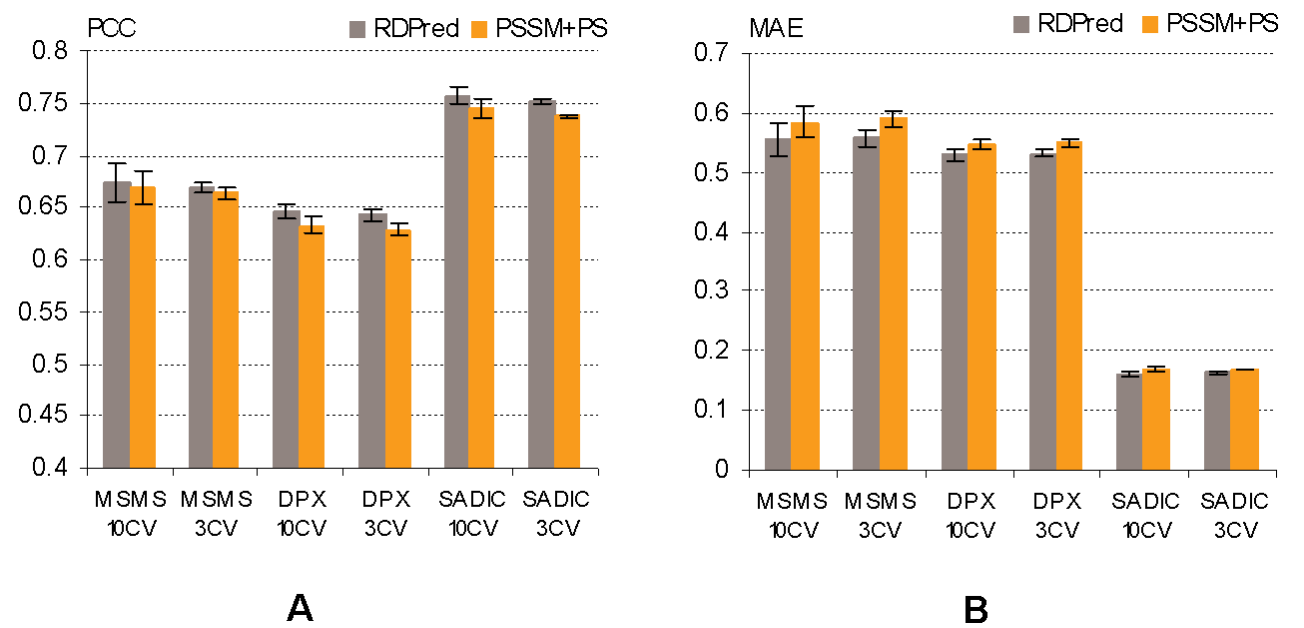
**The comparison (A) PCC and (B) MAE values at the fold level using three-fold cross validations (3 CV) and ten-fold cross validations (10 CV) for the three depth indices, i.e., MSMS, DPX and SADIC, on the YW923 dataset. **The *x*-axis shows the depth index and test types, e.g., MSMS 10 CV corresponds to the results for the MSMS based depth derived by ten-fold cross validation. The results are averaged over the folds and the corresponding standard deviations are shown using error bars. The scale of the *y*-axis, which shows the average quality index values, varies between the two panels.

We applied the same features and parameterization as in the original RDPred method (developed for MSMS based depth) and performed three-fold and ten-fold cross validations on the YW923 dataset using the other two depth indices. We tested two sets of features, PSSM+PS (which simulates the YW method) and RDPred's features. The prediction quality measured with PCC and MAE at the residue level is summarized in Table 7 while the PCC and MAE values at the fold level are visualized in Figure 7. We did not compute the MRE values because the two other depth indices include values of zero. The MAE values and the corresponding centiles shown in the Table 7 should not be compared across different depth indices due to large differences in the range of values for the three depth indices.

**Table 7:** Comparison of the prediction performance at the residue level between RDPred and simulation of YW method (using PSSM + PS features) when using three different depth indices.

		MSMS		DPX		SADIC	
		
Test type	Prediction method	PCC	MAE	PCC	MAE	PCC	MAE
3 CV	PSSM+PS	0.66	0.59 (0.049,1.502)	0.63	0.55 (0.060,1.305)	0.74	0.17 (0.023,0.367)
	RDPred	0.67	0.56 (0.037,1.510)	0.64	0.53 (0.054,1.308)	0.75	0.16 (0.021,0.360)

10 CV	PSSM+PS	0.67	0.58 (0.048,1.496)	0.63	0.55 (0.058,1.302)	0.74	0.17 (0.023,0.363)
	RDPred	0.67	0.56 (0.036,1.501)	0.65	0.53 (0.053,1.307)	0.76	0.16 (0.021,0.358)

The results are based on three-fold and ten-fold cross validations on YW923 dataset. The values in the brackets correspond to the 10^th ^and 90^th ^centiles of the absolute errors.

In general, the results suggest that all considered depth indices can be accurately predicted from the sequence. The PCC values for the DPX index are similar to values obtained for the MSMS based depth, which is due to high correlation between these two depths values and the fact that both definitions are based on the distance. As shown in Figure 7, MSMS based depth has bigger error bars for both PCC and MAE values when compared with the other two depth indices. This is likely due to the fact that MSMS index is characterized by the largest standard deviation of the depth values, i.e., the standard deviation equals 1.41, 1.05, and 0.33 for MSMS, DPX, and SADIC indices, respectively. We performed the paired t-test for PCC and MAE values at the fold level for each depth index that compares PSSM+PS method and RDPred. These t-tests were based on ten-fold cross validations on the YW923 dataset. For the MSMS, DPX, and SADIC based depth definitions the corresponding P-values equal 0.19, 9.2E-7, and 2.6E-7 for PCC, and 1.8E-10, 2.5E-9, and 1.4E-8 for MAE, respectively. We observe that statistically significantly better PCC values were obtained for the DPX and SADIC indices, while the difference for the MSMS index is not significant. At the same time, using the proposed method to predict each of the three depth indices results in statistically significant improvements of the MAE values.

**Figure 8:**
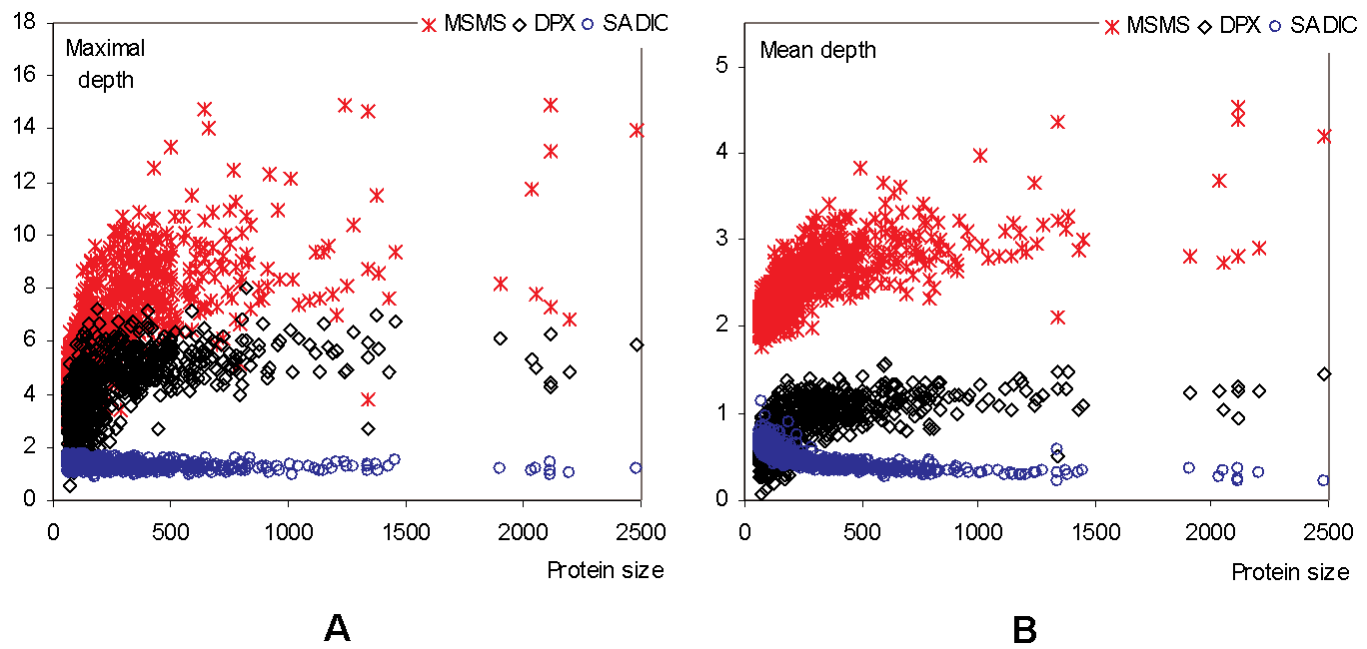
Relation between protein size and (A) maximal depth and (B) mean depth for the three depth indices, i.e., MSMS, DPX, and SADIC.

**Figure 9:**
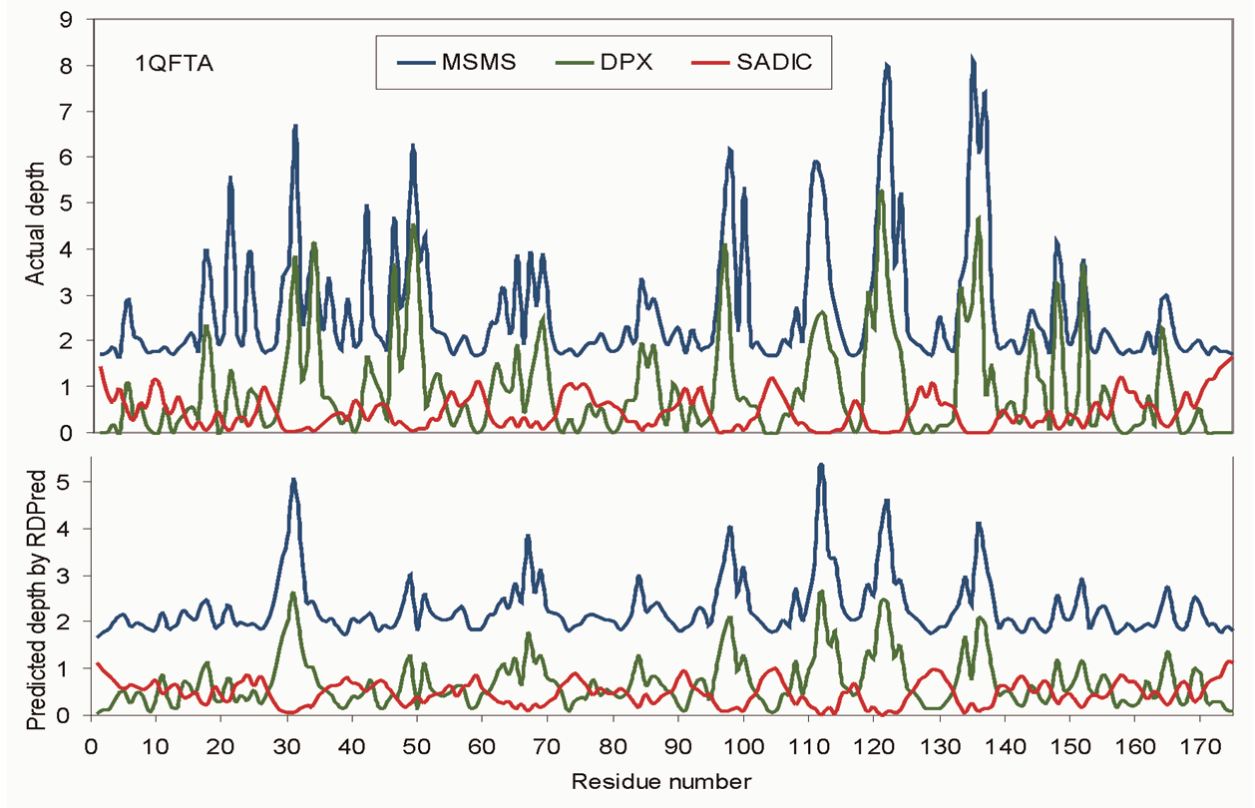
**The comparison of the observed depth values (the top plot) and the predicted depth values (the bottom plot) for 1QFTA protein chain.** Blue, green, and red plots correspond to actual and predicted MSMS, DPX, and SADIC depth values.

Following we analyze why the depth defined with SADIC algorithm yields higher quality predictions. The reason for the differences in the prediction performance between SADIC and the other two depth indices is due to the fact that SADIC is a volume based depth, while the other two are distance-based. The volume based depth is negatively correlated with the other two distance based depth indices. As shown in Figure 8, the maximal and mean distance based depth values (defined with MSMS or DPX) show larger variability with the increasing size of the protein chain, while the maximal and mean volume based depth values are independent of the chain size. The definitions of the three depth indices imply that the volume based depth has an upper bound of 2 while both distance based indices have no upper bound. The prediction of the indices that are characterized by larger variability and wider range of values is more challenging. The wider range of values implies that the corresponding MAE values (which are not normalized against the range) are higher. The reason for the improved PCC values in the case of the SADIC depth index is that these depth values along the protein sequence (referred to as the depth profile) are smoother. On the other hand, the other two indices are characterized by larger number of high spikes, see Figure 9, in which case it is harder to generate highly correlated values. To show that, we counted the number of spike points for each depth index for sequences from the YW923 dataset. Residue *i *is called an *ε*-spike point if its depth satisfies

(RD_*i*_-RD_*i*+1 _<*ε *and RD_*i*_-RD_*i*-1 _<*ε*) or (RD_*i*_-RD_*i*+1 _> *ε *and RD_*i*_-RD_*i*-1 _> *ε*)

where RD_*i *_is the distance or volume based depth of the *i*^th ^residue in the sequence and *ε *measures the magnitude of the spike with respect to the adjacent residues.

Table 8 shows the counts of spike points in the YW923 dataset for values of *ε *ranging between 0 and 1.0 with step of 0.1. For each given value of *ε *the volume based depth profiles have the smallest number of spikes. The number of spike points for SADIC based depth decreases sharply with the increase of the *ε *value and no *ε*-spikes are found for *ε *≥ 0.5. At the same time, a large number of spikes with *ε *≥ 1.0 can be found for both distance based depth profiles. We visualize the abovementioned differences using the actual and the predicted depth profiles for the 1QFTA protein (the same protein was used in Figure 6A), see Figure 9. The predictions were performed with RDPred using three-fold cross validation test. The MAE values for MSMS, DPX, and SADIC based depth values are 0.56, 0.52, and 0.17, respectively, and they are close to the average prediction quality shown in Table 7. Similarly, the corresponding PCC values equal 0.73, 0.71 and 0.82, respectively. The profiles of the two distance based depth indices are similar and they show a relatively large number of high magnitude spikes, which are under-predicted by RDPred. This is in contrast to the volume based depth profile that is smoother and which includes a smaller number of lower magnitude spikes.

**Table 8:** The number of spike points in the function of *ε *values for the three considered depth indices on the YW923 dataset.

	# of spike points		
	
*ε *value	MSMS	DPX	SADIC
0.0	92883	123137	79386
0.1	66195	108918	39436
0.2	49618	92639	15922
0.3	39447	80172	3416
0.4	32388	69112	133
0.5	27414	59932	0
0.6	23847	51979	0
0.7	21120	45250	0
0.8	18890	39300	0
0.9	17092	34494	0
1.0	15487	30082	0

Although we observe improvements for all three depth indices when comparing predictions of the proposed RDPred and when using PSSM+PS features, we emphasize that the results for the DPX and SADIC depth indices could be potentially further improved if a separate feature set and SVR parameterization would be performed (which is outside of the scope of this contribution). We also stress that this discussion should not be used to evaluate which of the indices is the best measure of the residue depth.

### Analysis of sequence representation

In this and the next sections, the analysis focuses on the MSMS based depth index. Table 2 shows that feature selection performed to design RDPred provided improvements with respect to the depth predictions. When comparing "All features" and RDPRed rows, we observe that the corresponding MAE and MRE values are lower when reduced feature set was used. At the same time, feature selection also allows analysis of the significant factors that influence prediction of the residue depth. Table 9 shows the number of original features and the number of selected features (total of 125 features were selected) organized by feature types (see "Feature Vector" section).

**Table 9:** The number of selected features for different feature types.

	PSI-BLAST-based		PSIPRED-based			Sequence-based	
Feature type	PSSM	IPP	SSP	Content	fseg	Protein size	Position
Total # of features	300	15	45	3	3	1	1
# of selected features	96	2	24	0	1	1	1

We divide our discussion of the selected features into four parts: PSSM-based features, PS (protein size), SS features (probabilities of secondary structure prediction, content and frequency of secondary structure segments), and PI features (position and information per position). First, we investigate the values added of each of these sets by comparing prediction quality of RDPred on the YW923 dataset when using all features with removing one of the above four sets at the time, see Table 10.

**Table 10:** Comparison of residue depth prediction quality when removing individual sets of features used by RDPred.

Feature sets used	PCC	MAE	MRE(%)
RDPred (all features)	0.668	0.558 (0.037,1.510)	17.0 (1.8,41.4)
PSSM removed	0.518	0.656 (0.041,1.907)	18.9 (2.1,47.4)
PS removed	0.632	0.575 (0.037,1.555)	17.3 (1.9,42.3)
SS removed	0.650	0.575 (0.037,1.556)	17.3 (1.9,42.4)
PI removed	0.664	0.562 (0.037,1.519)	17.1 (1.8,41.6)

The tests were performed on the YW923 dataset using three-fold cross validation. The values in the brackets correspond to the 10^th ^and 90^th ^centiles of the absolute and relative errors.

As shown in Table 10, the largest decrease by 0.15 for PCC and the largest increases by 0.1 and 1.9 for MAE and MRE, respectively, result from removing PSSM features. This suggests that the multiple alignments computed with PSI-BLAST that reflect evolutionary information provide the highest quality input for regression-based prediction of residue depth. On the other hand, the prediction quality remains relatively high when PSSM features are removed, which implies that the remaining features computed based on the predicted secondary structure and sequence also provide substantial amount of useful information. Moreover, comparable values of MAE and MRE are obtained in case of removing the PS and SS features. This shows that protein size and predicted secondary structure provide similar amounts of improvement. Large improvements related to adding the protein size to the PSSM features that were reported in [30] support the claim that our design constitutes a step towards providing an accurate sequence-based depth prediction method. Finally, the smallest improvements are associated with PI features.

**Figure 10:**
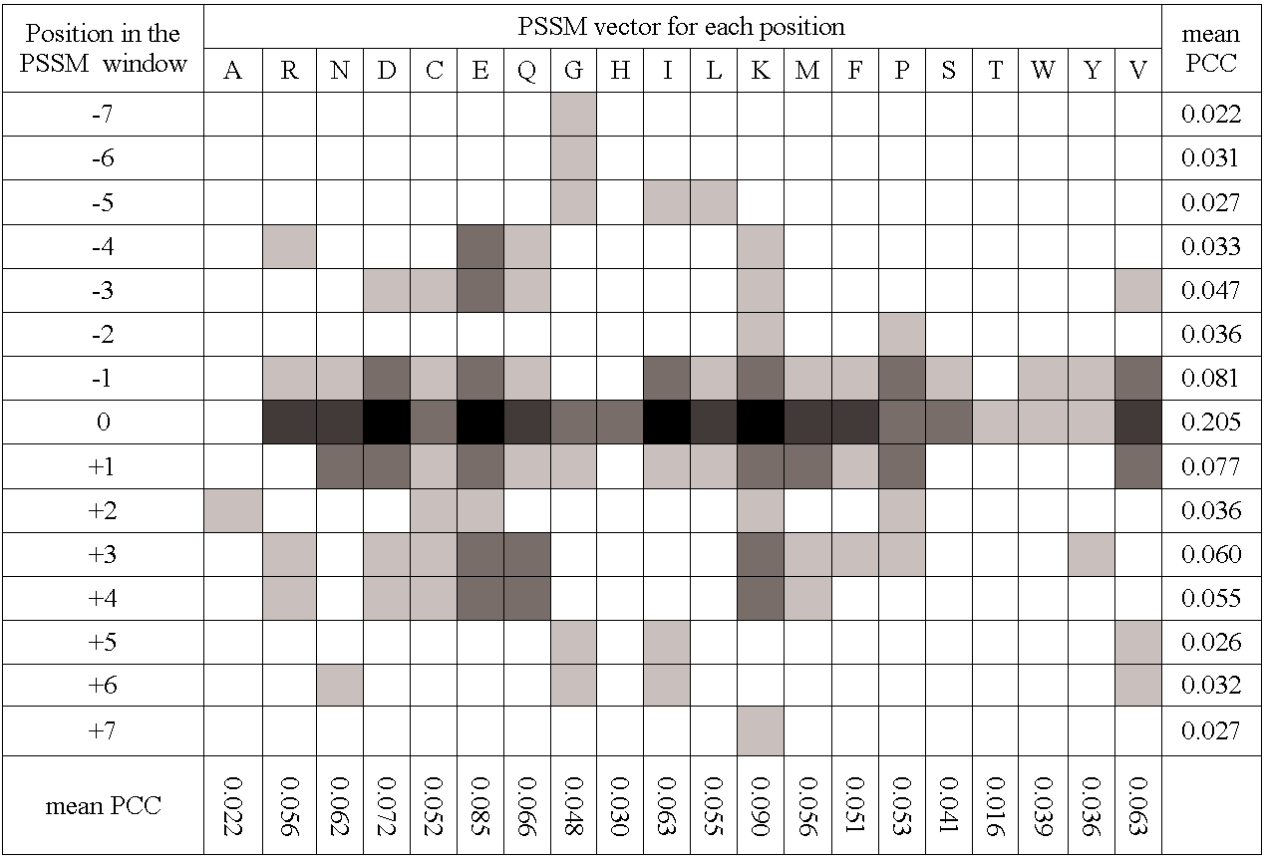
**Distribution of PSSM features ranked based on PCC values of the corresponding, selected features.** The shading of the individual cells (features) corresponds to their absolute average (over 3 folds in YW923 dataset) PCC values: black for PCC ≥ 0.3, dark gray for 0.2 ≤ PCC < 0.3, gray for 0.1 < PCC ≤ 0.2), light gray for the remaining selected features, and white for features that were not selected.

The majority of RDPred's features (96 out of 125) correspond to PSSM values. At the same time, only 96 out of the total of 300 PSSM values were selected. Figure 10 visualizes the distribution of the selected PSSM features with respect to their positions in the window. Each cell in the Figure corresponds to one PSSM based feature and the shades of gray represents the absolute average PCC values (the value used during feature selection), i.e., darker color corresponds to stronger correlation, while white color shows which features were not selected. We also computed the mean PCC values for each row and column in Figure 10. As expected, the distribution of PCC values is relatively symmetric with respect to the central position in the window, which implies that the residues at the N-termini and C-termini in the local window have similar impact on the prediction performed for the central residue. The central residue (denoted by position 0) has the largest mean PCC, while other positions are characterized by lower mean PCC values. In general, the mean PCC values decline with the increasing distance from the central position. One exception are positions -2 and 2 (two residues away from the central residue) in which case the mean PCC is smaller than that for positions +/- 1 and +/- 3. While it is obvious that the positions immediately adjacent to the predicted residue should have large impact on the prediction, we hypothesize that the low average PCC value for the +/- 2 positions is due to limited interactions between these residues and the central residue. For instance, in case of helical structure, the residues at +/- 3 or +/- 4 positions would form hydrogen bond with the central AAs, in contrast to the residues at +/- 2 position. The strongest PCC values for +/- 3 or +/- 4 positions are observed for Glu (E), Gln (Q), and Lys (K), and these residues were shown to be strongly associated with the formation of helices [62], which further substantiates our hypothesis.

The features corresponding to Glu (E), Lys (K), Asp (D), and Ile (I) for the central residue have the largest PCC values. Moreover, the mean PCC values averaged over the window for different AAs show variations. As presented in Figure 10, Glu (E), Lys (K), and Asp (D) have the largest mean PCC. According to Table 5, these three AAs are charged, hydrophilic, and the most flexible. We observe that in general, PSSM values of hydrophilic AAs are more strongly correlated with the residue depth than hydrophobic AAs, aliphatic amino acids (Val (V), Ile (I), and Leu (L)) have greater mean PCC values than aromatic AAs (Phe (F), Ser (S), Trp (W), and Tyr (Y)), and finally the tiny AAs (Gly (G), Ser (S), and Tyr (Y)) are also characterized by small mean PCC values.

Table 11 shows the PCC values (and the corresponding rank) of the remaining selected features, which include the predicted secondary structure based features, and PS and PI features. In case of the secondary structure probability profiles, we note that the features derived from helices were not included. This is due to relatively uniform distribution of helices with respect to their depths in the protein when compared with the distributions of strands and coils, which had been shown in [30]. Yuan and Wang show that strand residues are biased towards the interior of the protein, while coil residues have a bias to be exposed, and thus the corresponding features were found to be correlated with the residue depth. Furthermore, similarly as in case of the PSSM features, we observe that the magnitude of the PCC values for secondary structure based features declines with the increasing distance from the central residue. In case of the six features based on the global (sequence-wide) content and segment frequency of the predicted secondary structures, only the coil segment frequency was selected. We hypothesize that this could be due to a relatively more "loosely packed" conformations for proteins with larger number of coil segments, where as a result the corresponding proteins would have relatively wider distribution of the residue depth values. Finally, we observe that protein size (PS) is characterized by relatively large PCC value, which confirms results in [30], and that IPP of the central residue and at +4 position are selected. We hypothesize that the latter is likely due to the formation of hydrogen bonds in helices.

**Table 11:** Summary of the selected features, with exception of the PSSM features.

Rank^a^	Feature	PCC	Rank	Feature	PCC	Rank	Feature	PCC
5	SSP_C_^0^	0.292	9	SSP_E_^0^	0.279	18	PS	0.225
11	SSP_C_^-1^	0.257	12	SSP_E_^-1^	0.254	34	IPP^0^	0.120
14	SSP_C_^+1^	0.251	17	SSP_E_^+1^	0.231	124	IPP^+4^	0.056
20	SSP_C_^-2^	0.178	22	SSP_E_^-2^	0.170			
21	SSP_C_^+2^	0.175	26	SSP_E_^+2^	0.144	80	Position	0.079
60	SSP_C_^-7^	0.095	53	SSP_E_^+6^	0.098			
68	SSP_C_^-6^	0.086	63	SSP_E_^+7^	0.089			
74	SSP_C_^+7^	0.081	70	SSP_E_^+5^	0.085			
78	SSP_C_^-5^	0.080	86	SSP_E_^-3^	0.077			
83	SSP_C_^-3^	0.079	108	SSP_E_^-6^	0.061			
84	SSP_C_^+6^	0.079	112	SSP_E_^-7^	0.059			
96	SSP_C_^+3^	0.069						
110	SSP_C_^+5^	0.061	62	fseg_C_	0.089			

^a ^the rank of a given feature among the selected 125 features; this excludes the features based on PSSM.

### Prediction accuracy for buried and exposed residues

**Figure 11:**
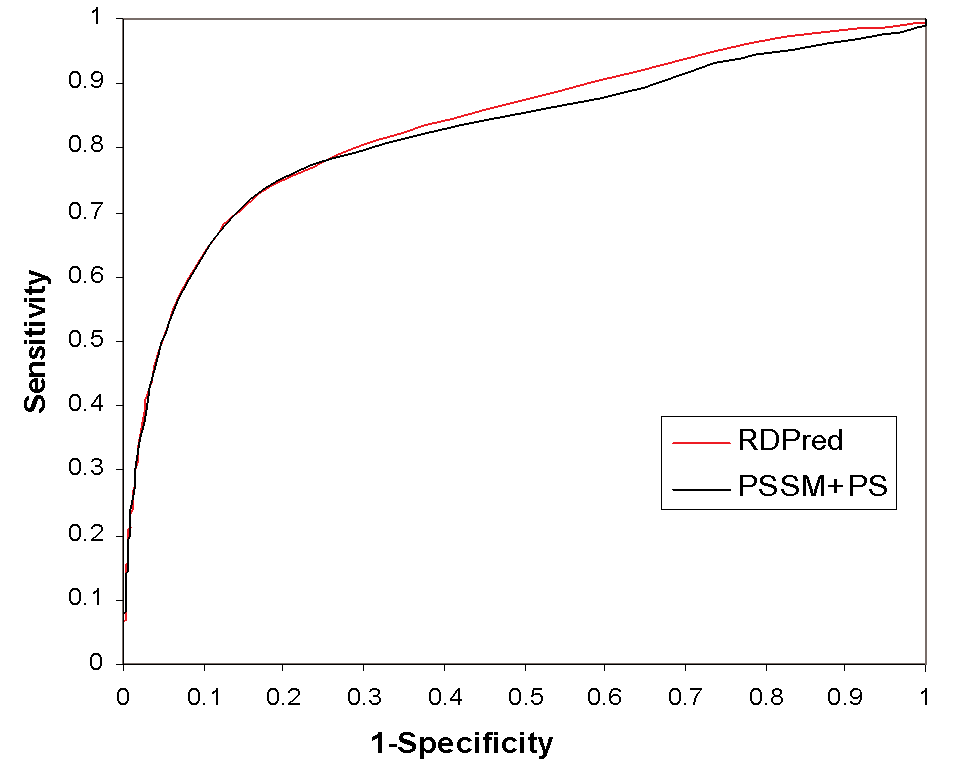
ROC-based comparison of two-state prediction when using the RDPred and simulation of YW method to predict residue depth, which is next binarized with a use of threshold to obtain the two classes.

One of the applications of the predicted residue depth is to distinguish between buried and exposed residues. We used Receiver Operating Characteristic (ROC) [63] analysis to investigate and compare the accuracy of the two-state (buried vs. exposed residues) prediction computed based on predicted residue depth. We choose a series of depth thresholds to classify the residues into the two classes and we compare the quality of the two-state prediction between the RDPred and the simulation of YW method (based on the PSSM and PS features). Figure 11 shows ROC plot of the TP rate (sensitivity) on *y*-axis against the FP rate (1-specificity) on the *x*-axis. The sensitivity is defined as the ratio of the number of correct predictions for buried residues to the total number of actual buried residues. Specificity is the ratio of the number of correctly predicted exposed residues to the total number of actual exposed residues. We observe that the RDPred provides on average better predictions when compared with the simulation of YW method. The improvements concern predictions with sensitivity of above 0.8 and specificity of below 0.7, i.e., the ROC curve for RDPred is above the ROC curve of the simulation of the YW method for these values.

Both, the pioneering work in sequence-based prediction of residue depths of Yuan and Wang [30] and our analysis show that it is more difficult to predict buried residues than the exposed residues when using predicted residue depth as the input. The main reason is that most of the residues are exposed (or close to the protein surface), see Figure 3, and thus the prediction methods are biased to improve these predictions, in contrast to less frequently occurring depth values for buried residues. Yuan and Wang suggested using the relative depth rank in the entire sequence to interpret the depth values, since this would allow finding deeply buried residues, which in turn would help when modeling structure of novel proteins [30]. To this end, the MSMS based depth values of a protein sequence were sorted in ascending order; this was applied to both the predicted and the observed depth values. When selecting the top 10% buried residues in each chain in YW923 dataset using both observed and predicted values, the resulting overlap (sensitivity within this set of residues) for the predictions with RDPred equals 49.8%. This ratio is better than the ratio reported for the YW method, i.e., 47.8%. Table 12 shows the ratios for increasing values of the top percentages. The table shows that RDPred is characterized by improved sensitivity when compared with YW method and the simulation of the YW method. The improvements gradually decrease as the threshold values increase, which is due to inclusion of exposed residues for the larger thresholds. We repeated this analysis but when considering the sensitivity of predicting the most exposed residues, see Table 13. We used a wider range of thresholds since majority of the residues are exposed, see Figure 3. The results again shows that the proposed RDPred method provide higher sensitivity of prediction of the exposed residues when compared with the simulation of the YW method. We conclude that the improvements in the residue depth prediction performed with RPPred result in improved ability to predict both the most buried and the most exposed residues.

**Table 12:** Sensitivity of the prediction of the top 10–35% of the most buried residues.

The most buried (%)	10	15	20	25	30	35
YW method	47.8	55.1	60.0	63.6	66.6	69.9
PSSM+PS	49.2	56.5	61.2	64.2	66.6	68.5
RDPred	49.8	57.1	62.3	65.3	67.8	70.1

**Table 13:** Sensitivity of the prediction of the top 10–90% of the most exposed residues.

The most exposed (%)	10	20	30	40	50	60	70	80	90
PSSM+PS	27.3	42.9	55.3	65.3	73.4	80.1	85.6	90.2	94.3
RDPred	33.0	48.6	59.6	68.3	75.4	81.2	86.1	90.5	94.4

## Conclusion

We propose an accurate method for sequence-based real-value prediction of residue depth. When compared with the recently proposed method by Yuan and Wang that utilized PSSM matrix and protein size as the input [30], the proposed RDPred method applies several new sources of information including predicted secondary structure, residue position, and information per position in the PSI-BLAST profile. We also perform feature selection that reduces the dimensionality of the input vector and allows for investigation into the relations between the input features and the predicted depth values. Our analysis shows that: (1) the most important new features, except the PSSM and protein size that have been studied in [30], are the features based on the predicted secondary structure; (2) hydrophilic and flexible residues are easier to predict than hydrophobic and rigid residues; (3) charged residues that include Lys, Glu, Asp, and Arg are the most accurately predicted; (4) the evolutionary information encoded using PSSM is characterized by stronger correlation with the depth for hydrophilic AAs and aliphatic AAs when compared with hydrophobic AAs and aromatic AAs; and (5) secondary structure of coils and strands is useful in depth prediction, in contrast to helices that are characterized by a more uniform distribution over their depth in the protein.

We investigated the quality of the prediction when considering two distance based, MSMS and DPX, and one volume based, SADIC, depth indices. The proposed RDPred method obtained 0.75/0.76 PCC at the residue level based on three-fold/ten-fold cross validation when predicting SADIC defined depth values, which is better than the correlations obtained for the other two depth indices, i.e., 0.67/0.67 and 0.64/0.65, respectively. This is likely due to the fact that the depth defined using a SADIC index is easier to predict since the corresponding depth profile along the protein chain is smother and it contains smaller number of spikes than the profiles of the other two indices. In general, our results suggest that all considered depth indices can be accurately predicted from the sequence.

The proposed method provides statistically significantly better predictions of MSMS based residue depth, i.e., the MRE and MAE values predicted by RDPred are significantly lower than the corresponding values predicted with the method by Yuan and Wang. Similar conclusions are drawn for the two other depth indices. We show that the predicted depth can be used to provide improved prediction of both buried and exposed residues when compared with the competing method. The prediction of exposed residues has implications in characterization/prediction of interactions with ligands and other proteins, while the prediction of buried residues could be used in the context of folding predictions and simulations. Both, the proposed and the competing method are characterized by higher quality of prediction for the exposed residues.

In conclusion, RDPred method constitutes a step towards providing an accurate sequence-based residue depth prediction method.

## List of Abbreviations

YW method: Yuan and Wang method; RDPred: residue depth prediction method; PSSM: position-specific scoring matrix; IPP: information per position; SSP: secondary structure probability profiles; PS: protein size; PI: position and information per position; PCC: Pearson correlation coefficient; MAE: mean absolute error; MRE: mean relative error; CV: cross validation; RSA: relative solvent accessibility; SVR: Support Vector Regression; ROC: Receiver Operating Characteristic; PDB: Protein Data Bank.

## Authors' contributions

HZ contributed to the conception and design of the prediction method, designed and computed the features, performed feature selection and experimental comparison, and helped with evaluation of the results. TZ contributed to the conception and design of the prediction method and helped with the evaluation of the results. KC helped with the preparation of the datasets and the experimental study. SS and JR contributed to the conception and design of the prediction method. LK contributed to the conception and design of the prediction method, helped with design of the experimental study and evaluation of the results, and coordinated the project. All authors have drafted, corrected and approved the manuscript.

## Supplementary Material

Additional file 1 **Supplementary Table 1.** List of PDB ids (including the chain identifier) of sequences from the PDB491 dataset Click here for file
