# Supplementary material for: Sequence based residue depth prediction using evolutionary information and predicted secondary structure
Source: BMC Bioinformatics. 2008 Sep 20;9:388. doi: 10.1186/1471-2105-9-388 (PMC2567998; doi:10.1186/1471-2105-9-388)
Supplement: Additional file 1 — Supplementary Table 1. List of PDB ids (including the chain identifier) of sequences from the PDB491 dataset [file 1471-2105-9-388-S1.doc]

**Supplementary Table 1.** List of PDB ids (including the chain identifier) of sequences from the PDB491 dataset.

| 2PZ4A  2PY2A  2HB0A  2OFQA  2J6IA  2QEDA  2NP9A  2PK0A  2HWTA  2OBDA  2PSDA  2JBWA  2HNLA  2UWAA  2IFGA  2DV6A  2CAPA  2IUEA  2DH2A  2Q7NA  2INPC  2OOGA  2UUEB  2ORXA  2OG4A  2Q3FA  2OBTA  2P12A  2PT2A  2QLTA  2QMOA  2ONSA  2OJHA  2PSTX  2QL3A  2PG0A  2QJVA  2OWNA  2QPXA  2PBLA  2QDRA  2QE8A  2Q04A  2P4OA  2P2SA  2O62A  2O1QA  2P4GA  2PYXA  2Q02A | 2OH1A  2Q14A  2QE6A  2QIWA  2PGCA  2P7IA  2OIKA  2OZGA  2OWBA  2Q0XA  2NTEA  2DSKA  2IKQA  2Q8PA  2OBLA  2UVJA  2IRUA  2V8PA  2NVOA  2ISKA  2HFSA  2P0WA  2DURA  2E61A  2ECLA  2E5RA  2PKFA  2DX8A  2HK9A  2IZ1A  2EI9A  2PUYA  2DW0A  2E7ZA  2INPA  2DFZA  2E3ZA  2PQYA  2DDXA  2NSUA  2PV0B  2PHZA  2IPIA  2P17A  2V27A  2Z20A  2NVVA  2O4UX  2JQXA  2HIHA | 2QS9A  2HI4A  2UWIA  2E9XB  2DC0A  2P3NA  2HZLA  2PQ5A  2OOXG  2Z1DA  2Z2NA  2OEGA  2JBRA  2OR7A  2OWOA  2JEXA  2JEPA  2EHHA  2IXBA  2OITA  2O5VA  2DTXA  2EBFX  2DHOA  2Q0ZX  2CF5A  2JE8A  2QNEA  2Q8NA  2ORDA  2DYUA  2OGEA  2OAUA  2O0JA  2D73A  2OA1A  2OSXA  2IU8A  2P4EP  2E11A  2JIFA  2UZ9A  2P8UA  2UYYA  2JFKA  2POKA  2QFLA  2Q0LA  2DVYA  2PHCB | 2OPIA  2IMFA  2OHHA  2GGOA  2DU3A  2FN9A  2JFZA  2P8BA  2OQYA  2JC4A  2GH9A  2P3YA  2JENA  2DTVA  2PO3A  2I2CA  2IVNA  2DQ0A  2O0MA  2JC5A  2DXNA  2E0ZA  2H3HA  2OWLA  2ODXA  2E2OA  2P0VA  2NQ2C  2C20A  2OASA  2UVEA  2P1MB  2PH1A  2GHAA  2O7IA  2JRBA  2HTAA  2H1YA  2O2PA  2UXYA  2O4CA  2P6PA  2E87A  2GGSA  2GT1A  2P5YA  2PJDA  2HPGA  2OEMA  2P90A | 2HKEA  2IBPA  2V3AA  2IXDA  2PJUA  2Q01A  2PB9A  2PMQA  2QGYA  2PA4A  2IHOA  2OZTA  2QEEA  2QAHA  2PGWA  2GBWA  2IMPA  2PUJA  2JG2A  2OLNA  2FR7A  2ODFA  2HLKA  2EABA  2HOQA  2CMGA  2O09A  2P7JA  2DTCA  2GMNA  2G5XA  2PE4A  2QNKA  2HA1A  2I33A  2OLGA  2OCEA  2OKTA  2OUXA  2POFA  2P4DA  2PA9A  2QM1A  2QMWA  2P3PA  2H4AA  2I99A  2OKJA  2PFZA  2JH1A | 2GFIA  2OH5A  2UWQA  2JC7A  2FMYA  2QCKA  2DD4A  2I50A  2DQAA  2Q03A  2NXPA  2P39A  2QGGA  2P62A  2OA2A  2UZGA  2QIPA  2GBWB  2PXXA  2HZQA  2QGUA  2OPKA  2DGDA  2PNLA  2PPWA  2EDMA  2JM1A  2OWAA  2IIFA  2E18A  2FQHA  2JA4A  2V82A  2QEAA  2E9XA  2OD4A  2QPWA  2ED6A  2OV9A  2P2EA  2OTDA  2OIWA  2ONFA  2OAFA  2GZBA  2UV0E  2PA7A  2PW4A  2P84A  2PRVA | 2QL8A  2P97A  2JPEA  2JDAA  2E44A  2PFWA  2O6LA  2QHQA  2OOXB  2HW0A  2DVJA  2OOKA  2OZHA  2OSTA  2FXTA  2OD6A  2V1OA  2I0NA  2PQVA  2Q9KA  2H1EA  2E4MC  2OWPA  2PHPA  2FHDA  2O03A  2JNQA  2J0EA  2PLGA  2DD4B  2ICNB  2V14A  2PKHA  2OB5A  2Q0YA  2OU5A  2P25A  2DLAA  2QKPA  2PLIA  2DJMA  2E56A  2O6PA  2J49A  2QNUA  2G37A  2HU9A  2OS5A  2QMMA  2OD5A | 2JMPA  2QF4A  2P13A  2GFUA  2HDEA  2PWWA  2PDOA  2INPE  2Q2GA  2PYTA  2E6IA  2EDOA  2GFFA  2DDZA  2OHWA  2PR1A  2NLWA  2FPHX  2NPLX  2OOXA  2P1JA  2Q5CA  2H09A  2PJSA  2E5IA  2OT9A  2OGGA  2CHCA  2OVSA  2OP5A  2I9SA  2ILLA  2JQ5A  2HO9A  2H36X  2PN0A  2I6JA  2HJQA  2NNCA  2H7TA  2H7AA  2OD0A  2HF6A  2DT8A  2EA5A  2CM4A  2OTMA  2E5SA  2E7CA  2E1BA | 2OX7A  2PZZA  2O3GA  2EC1A  2DU9A  2P5VA  2GXGA  2JN9A  2P3HA  2EBWA  2QG3A  2O38A  2G3WA  2JOZA  2IBLA  2E5GA  2QH0A  2OXGZ  2E29A  2E6JA  2DZKA  2NSCA  2E67A  2V1YB  2PG4A  2OUTA  2IVWA  2V0FA  2E63A  2E5NA  2EBKA  2PJHA  2P5TA  2OYZA  2V1YA  2Q78A  2HW2A  2GJ3A  2HSXA  2DK6A  2FK5A  2PIMA  2J6BA  2DPWA  2DTJA  2ODKA  2E70A  2CNTA  2OX6A  2GS9A | 2QJWA  2DZJA  2PK8A  2V3SA  2JNGA  2JODA  2JNAA  2HTJA  2JN0A  2E0GA  2JE0A  2HQLA  2OPCA  2DYIA  2QQZA  2DM9A  2QRRA  2Q30A  2JOEA  2P19A  2JRRA  2QGPA  2JOYA  2P4WA  2E7MA  2OA4A  2OZJA  2E6ZA  2PD0A  2YSUB  2DSYA  2Q7BA  2PC1A  2ICIA  2E5PA  2JQOA  2QSIA  2PD1A  2DX6A  2P4PA  2JSCA |
| --- | --- | --- | --- | --- | --- | --- | --- | --- | --- |

**Supplementary Table 2.** List of PDB ids (including the chain identifier) of sequences from the PDB366 dataset.

| 2JBRA  2I33A  2PJUA  2PB9A  2G3WA  2NXPA  2NNCA  2EABA  2JE8A  2EBFX  2D73A  2E7ZA  2UVEA  2P1MB  2DU3A  2IPIA  2OEGA  2OKJA  2QNEA  2P3YA  2Q7NA  2OSXA  2POKA  2OBDA  2Q8NA  2IXBA  2QEEA  2OASA  2OITA  2JFKA  2DH2A  2UVJA  2OLNA  2OZGA  2JBWA  2O0JA  2P6PA  2O4CA  2CAPA  2QPXA  2IU8A  2Z1DA  2HB0A  2HZLA  2O5VA | 2E87A  2E0ZA  2O0MA  2PJDA  2QE8A  2UXYA  2Q0ZX  2P2SA  2Q0XA  2DYUA  2O4UX  2DDXA  2OZTA  2IVNA  2INPC  2GT1A  2H4AA  2P90A  2ONSA  2I99A  2DSKA  2PHZA  2OAUA  2P4OA  2IRUA  2OWLA  2QDRA  2PFZA  2E2OA  2Z2NA  2OJHA  2IHOA  2P7JA  2OUXA  2P17A  2QLTA  2V27A  2DXNA  2QE6A  2I2CA  2Q02A  2IKQA  2O62A  2P4GA  2QJVA | 2QMWA  2E11A  2E67A  2CMGA  2OWNA  2PBLA  2PH1A  2Q8PA  2ODFA  2QIWA  2OG4A  2DURA  2P7IA  2OH5A  2JH1A  2PQYA  2P62A  2PZ4A  2DHOA  2DPWA  2ISKA  2POFA  2DVYA  2QNUA  2PHCB  2DGDA  2PD0A  2DLAA  2QMOA  2E1BA  2OV9A  2PPWA  2PXXA  2IUEA  2OPIA  2GS9A  2Q04A  2QGUA  2NTEA  2P3PA  2PGCA  2IMFA  2PNLA  2P4WA  2P97A | 2PC1A  2FK5A  2DM9A  2QMMA  2QS9A  2DDZA  2O09A  2PW4A  2JNQA  2E9XB  2Q3FA  2Q7BA  2OH1A  2DTJA  2P12A  2QJWA  2OU5A  2UV0E  2CHCA  2E63A  2ED6A  2O6LA  2V1YB  2HO9A  2OX6A  2FPHX  2QIPA  2PLGA  2PR1A  2EDMA  2I6JA  2O6PA  2CNTA  2QEAA  2P5TA  2DD4B  2H09A  2OX7A  2OIKA  2OTMA  2PQVA  2FHDA  2OB5A  2PRVA  2Q0YA | 2Q78A  2OGGA  2OAFA  2OSTA  2Q9KA  2QKPA  2CM4A  2E9XA  2HF6A  2JE0A  2QPWA  2HDEA  2J49A  2OA2A  2PKHA  2E4MC  2JDAA  2O1QA  2P84A  2E56A  2PDOA  2HW2A  2QL8A  2OZHA  2UWIA  2JM1A  2PA7A  2PIMA  2PN0A  2JPEA  2ONFA  2JOEA  2OWAA  2QH0A  2OOXA  2QSIA  2OIWA  2JOZA  2YSUB  2GFUA  2V14A  2OHWA  2PYTA  2OUTA  2HU9A | 2IBLA  2DU9A  2OWPA  2EBKA  2JQ5A  2P2EA  2OOKA  2OPCA  2PWWA  2DD4A  2DTCA  2I50A  2P25A  2QQZA  2EC1A  2QHQA  2DQAA  2E5IA  2EDOA  2O38A  2PJSA  2E7CA  2ICNB  2INPE  2JSCA  2OVSA  2OP5A  2HSXA  2OD5A  2PFWA  2HW0A  2OR7A  2OZJA  2E7MA  2IVWA  2E6JA  2H36X  2OPKA  2QGPA  2HJQA  2H7AA  2HQLA  2OD6A  2Q30A  2DZKA | 2FQHA  2J6BA  2NSCA  2JQOA  2V1YA  2E0GA  2JMPA  2DJMA  2GFFA  2JODA  2JN9A  2JNGA  2NLWA  2OD0A  2JNAA  2PD1A  2PK8A  2DK6A  2JA4A  2OA4A  2OD4A  2P3HA  2QRRA  2E5NA  2E5SA  2EBWA  2I9SA  2OOXB  2UZGA  2E44A  2JOYA  2NPLX  2V3SA  2OFQA  2PG4A  2E5GA  2HWTA  2OYZA  2E29A  2PLIA  2P13A  2ODKA  2DZJA  2JRBA  2DSYA | 2V0FA  2P4PA  2UWQA  2HTJA  2I0NA  2ODXA  2PJHA  2PSTX  2PV0A  2NSFA  2IUMA  2PSOA  2QNGA  2QPVA  2PN2A  2QFEA  2OKQA  2OBAA  2PLNA  2PR7A  2QKHA  2DUNA  2DSTA  2E7GA  2PEBA  2OQGA  2E6QA  2EBUA  2OJLA  2OXGA  2HH2A  2EBEA  2DHXA  2JNEA  2E5JA  2GTJA  2JS3A  2OBPA  2DYJA  2E5KA  2POIA  2JOQA  2EAQA  2JMJA  2JN4A | 2OO2A  2JOVA  2GMOA  2GJFA  2Z1CA  2DK7A |
| --- | --- | --- | --- | --- | --- | --- | --- | --- |
